# Entropy Could Quantify Brain Activation Induced by Mechanical Impedance-Restrained Active Arm Motion: A Functional NIRS Study

**DOI:** 10.3390/e24040556

**Published:** 2022-04-15

**Authors:** Byeonggi Yu, Sung-Ho Jang, Pyung-Hun Chang

**Affiliations:** 1 Department of Robotics Engineering, Graduate School, Daegu Gyeongbuk Institute of Science and Technology, Daegu 42988, Korea; ybg5437@dgist.ac.kr; 2 Department of Physical Medicine and Rehabilitation, College of Medicine, Yeungnam University, Daegu 42415, Korea; belado@med.yu.ac.kr

**Keywords:** brain activation, entropy, mechanical impedance, fNIRS, signal amplitude, beta value

## Abstract

Brain activation has been used to understand brain-level events associated with cognitive tasks or physical tasks. As a quantitative measure for brain activation, we propose entropy in place of signal amplitude and beta value, which are widely used, but sometimes criticized for their limitations and shortcomings as such measures. To investigate the relevance of our proposition, we provided 22 subjects with physical stimuli through elbow extension-flexion motions by using our exoskeleton robot, measured brain activation in terms of entropy, signal amplitude, and beta value; and compared entropy with the other two. The results show that entropy is superior, in that its change appeared in limited, well established, motor areas, while signal amplitude and beta value changes appeared in a widespread fashion, contradicting the modularity theory. Entropy can predict increase in brain activation with task duration, while the other two cannot. When stimuli shifted from the rest state to the task state, entropy exhibited a similar increase as the other two did. Although entropy showed only a part of the phenomenon induced by task strength, it showed superiority by showing a decrease in brain activation that the other two did not show. Moreover, entropy was capable of identifying the physiologically important location.

## 1. Introduction

Brain activation has increasingly attracted attention as a means to understand brain-level events in response to cognitive tasks or physical tasks [1,2,3,4,5], and studies of brain activation continue to increase [6]. Moreover, brain activation is one of the promising biomarkers that is necessary to establish the relationship between brain measures and clinical outcomes [7]. In addition, the procedures for processes such as fMRI or fNIRS using brain activation have been standardized and widely used [8,9,10,11,12]. However, its quantification still remains an issue [13]. 

So far, there have been two major means to quantify brain activation [13]: *signal amplitude* [14,15,16] and *beta value* [17,18]. Signal amplitude, often found in neuroimaging techniques associated with devices such as fMRI, fNIRS, or PET, has been widely used for its quantification, because its increased signal change is known to reflect the activation of a greater number of neurons [19]. Nevertheless, signal amplitude is determined by the *balance* of excitation/inhibition of neurons, rather than the actual number of activated neurons [20]. For instance, if decreased signal amplitude is observed, it may be the result of either decreased excitation, or increased inhibition, or both. This inability of signal amplitude to distinguish between excitation and inhibition has led some research using signal amplitude to derive inconsistent outcomes [20]. There was even a case where negative signal changes were observed for spontaneous limb movements [21]. In addition to balance, input synchrony is known to play a significant role in determining signal amplitude [22,23]. Because of this alarming nature, therefore, it was argued that signal amplitude should not be viewed as a measure of neuronal activation [24].

Beta value, which is applied to *t statistics* or *F statistics*, is usually regarded as the degree of compatibility with hemodynamic response function (HRF). Hence, it may be considered a value associated with the predictability of the measured signal [25]. Currently, the most widely used canonical HRF is modeled as a linear time invariant (LTI) system. Although it has a simple form, it is widely used [3,26,27]. It has been reported that canonical HRF captures the shape of the hemodynamic response (HDR) better than FIR (finite impulse response) or Fourier sets that are flexible [28]. However, there are also disadvantages. The model, being a function of time *only*, assumes no spatial variations across brain regions [28]. The *real* HDR, however, has been reported to vary across brain regions and subjects [29]. Further, it exhibits hysteresis [30], a form of nonlinearity, the presence of which makes a system nonlinear, not allowing *superposition* [31] necessary for the convolution of the HRF and stimuli. Therefore, in quantifying brain activation, beta value has its own limitations, deriving from the canonical HRF, which is the basis function to which the compatibility is scaled.

In order to cope with the inherent limitations of the LTI model, there have been attempts to improve HRF to encompass nonlinearities [32] and regional variation [33]. Unfortunately, however, finding such a precise and robust HRF has turned out to be an ill conditioned problem [34], making the LTI model the most common choice [35,36]. 

It is noteworthy that beta value, being used as a scaling factor to equalize the measured signal with the design matrix, which is the convolution of the HRF with the stimulus, may essentially be regarded as a form of signal amplitude [37]. Also, beta value, like signal amplitude, is placed in the context of regional signal variation that is affected by a task. Naturally, beta value shares the aforementioned downside of signal amplitude [28].

Apart from the above approaches, there have been attempts to evaluate brain activation using *entropy*, a thermodynamic entity. Since neuroimaging techniques measure the energetic activity of the brain [38], the presence of brain activation due to tasks could be detected by using the entropy of the measurement signal [39]. Also, the sequence of brain regions activated by a given task could be assessed by entropy [40,41], and altered states of consciousness could be distinguished by evaluating brain activation as entropy [42,43]. Further, in previous studies [41,44], entropy was used to represent the strength of brain activation. The study [44] was mainly concerned with the difficulty level of arithmetic calculation, which was evaluated by measuring the degree of brain activation in terms of entropy value. The research in [41] has used the causal links and relations of entropy to identify the sequence of activation as it is propagated from one part of a brain region to another, thereby determining the causality and connectivity of parts in the region; clearly, entropy was used as a marker of brain activation. However, while brain activation was represented by the entropy value in these studies, it was done intuitively without any justification, leaving the fundamental question of whether entropy is a relevant measure to represent brain activation unanswered. To our knowledge, there has been no research to address that question. In this article, therefore, we attempt to show that entropy is indeed a relevant measure for brain activation by providing appropriate evidence.

Since we desire to observe subtle changes in brain activation level, we consider it imperative to be able to differentiate the strength of the stimuli. To this end, we have adopted the mechanical *impedance*, a general resistance exerted by the robot to the subject. Until now, there have been studies using mechanical stimuli, such as displacement amplitude, velocity, and force to provide quantified stimuli [14,45,46]. However, there has been no study to employ mechanical impedance as the stimuli for brain activation. Mechanical impedance consists of inertia, damping, and stiffness; and is an important entity that characterizes the relationship between a task’s force and motion properties—position, velocity, acceleration—having far more comprehensive substance than the conventional mechanical stimuli [47]. In order to differentiate the stimuli, we have gradually changed the values of the two parameters representing mechanical impedance: natural frequency and damping ratio. Various combinations of these two parameters could produce required stimuli through the robot. In addition, we provided the combinations in *random* order, making it unpredictable to subjects which impedance condition would be applied, so that the stimuli would always be unfamiliar to them. 

In order to test the relevance of entropy as a quantification measure, we have investigated the following points. First and foremost, we have examined if entropy can indicate the *difference* between the task state and the rest state. This capability was regarded as a minimum, bottom-line requirement for a measure because no significant differences mean its incapability to discern even the most obvious activation difference—the difference between the condition with the presence of stimuli and that without them at all. Furthermore, that incapability would make it meaningless to strive to *differentiate* the stimuli between those two states. After confirming that capability, therefore, we varied the stimuli intensity. More specifically, we varied task intensity, which consists of task duration and task strength, in order to observe how entropy changes and how its changes reflect brain activation. Note that task strength represents the strength to overcome the imposed mechanical impedance while performing a given task. To summarize the task to the subject, we provided nine impedance conditions to each subject in random order, and the subject was able to experience the given impedance condition by providing the active extension-flexion motion of the elbow to the robot. In addition, tasks were performed in a paradigm in which nine 30-s rest period blocks, and nine 30-s task period blocks, were alternately provided. In conjunction, we evaluated both the signal amplitude and the beta value in order to compare these with entropy; as well as identified the location of activation, based on entropy, in order to relate this to the conventional brain activation map constructed from Statistical Parametric Mapping.

## 2. Methods

### 2.1. Overall Configuration

Our investigation consisted of three parts: stimuli generation, measurement, and data analysis (Figure S1). Specifically, we generated stimuli to provide to the subject; measured brain activation evoked by the stimuli; and, using the measured data and computed entropy, we performed statistical analysis, together with additional computations and analyses, to compare with competing methods using signal amplitude and beta value. 

The three parts have their own subdivisions. First, the stimuli generation part consists of a robot system, impedance controller, and visual feedback system. Second, the measurement part comprises functional near-infrared spectroscopy, subject, and experiment protocol. Third, the data analysis part is composed of data processing, entropy calculation, and statistical analysis. That part also encompasses the computations and statistical analyses of signal amplitude and beta value, respectively, as well as the transformation of beta value into a brain activation map. The details of these follow.

### 2.2. Stimuli Generation

#### 2.2.1. Robot System

The robot system in Figure S2a–c, developed in our laboratory, is an elbow joint robot that presents impedances as subjects make active movements, producing stimuli to the brain. With clinical relevance in mind, the robot was designed for stroke patients, has a torque capability of 59.78 Nm [48], exceeding by far the torque requirement, 44.15 Nm [48], of patients with the severest stiffness and even farther than that of typical males, 39 Nm [49]. Details of hardware specifications are in [48]. 

We used an industrial PC to operate the robot. The SENSORAY Model 626, a multifunction board, was used to receive the torque sensor signal and encoder signal, and send the computed control signal to the servo driver (Figure S2d). The program for the operating robot is written in C++ under Linux (Ubuntu 16.04, and kernel 4.9.80) with a real-time application interface (RTAI) [50]. The sampling time was 0.002 s.

#### 2.2.2. Control Method

To provide accurate and precise mechanical impedance, we used a control law that combines time delay estimation (TDE) and ideal velocity feedback (IVF) [51]. The TDE enables both to eliminate robot dynamics—continuous dynamics—and save its huge amount of computation; the IVF helps overcome inaccuracy due to discontinuous frictions. While the TDE and the IVF strive to eliminate both continuous dynamics and the effects of discontinuous frictions, the control law inserts the desired impedance. The mathematical details are provided as follows.

The control law combining TDE and IVF is expressed as follows:

(1) $$\begin{array}{ll} \tau \;= & \underset{TDE}{\underset{︸}{\tau_{t-L}\;-\;\bar{M}{\ddot{\theta }}_{t-L}}}\\ & +\;\underset{IVF}{\underset{︸}{\bar{M}\Gamma ({\dot{\theta }}_{\mathrm{ideal}}-\;\dot{\theta })}}\\ & +\;\underset{\mathrm{Desired}\;\mathrm{impedance}\;\mathrm{dynamics}}{\underset{︸}{\bar{M}\{{\ddot{\theta }}_{d}\;+\;M_{d}^{-1}{[\tau }_{\mathrm{measured}}\;+\;\tau_{\mathrm{preload}}\;+\;K_{d}{(\theta }_{d}-\;\theta )\;+\;B_{d}({\dot{\theta }}_{d}-\dot{\theta })]\}}} \end{array}$$

where $\tau \;{,\tau }_{\mathrm{measured}},\;\tau_{\mathrm{preload}}$: Actuator torque, measured torque from torque sensor, and preloaded torque (5 Nm), $\;{•}_{t-L}$: Time delayed value of  •, L: Control loop sample time (=2 ms), $\bar{M},\;\Gamma$: Positive definite diagonal gain matrices, $\theta_{d},\;\theta$: Equilibrium trajectory (= 0° or initial position), actual trajectory, ${\dot{\theta }}_{\mathrm{ideal}}$: ideal velocity ($≜\;\int \{{\ddot{\theta }}_{d}\;+\;M_{d}^{-1}[\tau_{\mathrm{measured}}\;+\;\tau_{\mathrm{preload}}\;+\;K_{d}(\theta_{d}-\theta )\;+\;B_{d}({\dot{\theta }}_{d}-\dot{\theta })]\}\mathrm{dt}$).

The desired impedance is specified in the following dynamics:

(2) $$\begin{array}{l} M_{d}({\ddot{\theta }}_{d}-\ddot{\theta })+B_{d}({\dot{\theta }}_{d}-\dot{\theta })+K_{d}(\theta_{d}-\;\theta )+\tau_{\mathrm{measured}}+\tau_{\mathrm{preload}}\;=\;0\\ \mathrm{or}\;({\ddot{\theta }}_{d}-\ddot{\theta })+2\omega_{n}\zeta ({\dot{\theta }}_{d}-\dot{\theta })\;+\;\omega_{n}^{2}(\theta_{d}-\;\theta )\;+\;M_{d}^{-1}(\tau_{\mathrm{measured}}\;+\;\tau_{\mathrm{preload}}\;)=\;0 \end{array},$$

where $M_{d}$: Desired mass, $B_{d}$: Desired damping constant, $K_{d}$: Desired spring constant, $\omega_{n}\;$: Natural frequency, ζ: Damping ratio.

The control gains in (1), tuned according to the guidelines outlined in the paper [51], were selected as $\bar{M}$ = 1.3 and Γ = 30 and used for a pilot test and experiments. To evaluate the results of impedance control, we have adopted Equation (3), the norm of the impedance error [51]:

(3) $$\left|s\right|\;=\;\left|\int {\{\ddot{\theta }}_{d}\;-\ddot{\theta }\;+\;M_{d}^{-1}{[\tau }_{\mathrm{measured}}{+\tau }_{\mathrm{preload}}{+K}_{d}{(\theta }_{d}-\theta )\;+\;B_{d}({\dot{\theta }}_{d}-\dot{\theta })]\}\mathrm{dt}\right|,$$

#### 2.2.3. Visual Guidance Feedback System

The visual guidance feedback system was used to provide subjects with both active motion guidance (the blue bar) and their present arm positions (red bar). See Figure S3a,b. The blue bar was made to oscillate every 3 s, from full flexion posture up to 30-degrees limit, based on a fifth order polynomial trajectory, offering subjects a visual cue for active extension-flexion motions (Figure S3c). Note that the fifth order polynomial trajectory is known as the optimal movement trajectory in a physiological aspect [52].

### 2.3. Measurement

#### 2.3.1. Functional Near-Infrared Spectroscopy(fNIRS) System

As a modality to measure the entropy of brain activation, we adopted fNIRS, which is widely recognized to be robust to motion artifacts [53], ecologically valid [53], and not affected by electrical artifacts [54], compared to fMRI, making it an attractive choice for use with the robot. 

When measuring brain activation with fNIRS, which normally uses hemoglobin (Hb) concentration change [55], we opted instead for its raw data: *optical density.* The latter not only can measure Hb concentration changes [56], but also has much higher bandwidth, and, thus, contains much richer high frequency content than the concentration change. Specifically, while the former is governed by hemodynamics, which have a bandwidth below 0.1 Hz [57,58], the latter has a bandwidth similar to EEG [59], around 0.5~50 Hz [60]. 

The fNIRS instrument used was SHIMADZU’s LABNIRS. The sampling rate in the experiment was set at 47.6190 Hz. The fNIRS data was transferred to industrial PC (Figure S4a) through UDP communication. Two types of data were transferred to the PC: the information data indicating the state of fNIRS, and the measurement data of brain activation. 

As to the optode positioning, we used whole-head fiber holders to secure the optode to the head of the subject according to the 10/20 international placement protocol. 

The optode positional information and the anatomical landmark positions (nasion, CZ, left and right pre-auricular points) of each subject were acquired by using FASTRAK, a 3D digitizer of Polhemus, after collecting optical density data. That information was processed through channel registration by using NIRS-SPM [11]. Figure S4b shows the channel average position and optode position of subjects in the Montreal Neurological Institute (MNI) space. Six source optodes (red circle) and six detector optodes (blue circle) were used and 17 channels were generated. 

#### 2.3.2. Subject Information

The subjects consisted of 23 applicants, 15 males and 8 females, with an average age of 20.96 and a standard deviation of 3.59. They were confirmed to be free of neurological or psychological diseases. One man’s data was excluded because the detector exhibited insufficient light reception, causing a poor signal-to-noise ratio. We received voluntary written agreements from all subjects, and the experiment was conducted under the permission of the DGIST Institutional Review Board (DGIST_160114-HR-003–06).

It is necessary to check if the *number* of subjects is sufficient for regression analysis. The heuristic guideline [61] supports the sufficiency. According to the guideline, when performing multiple regression analysis, 15 to 20 observations are required per independent variable. In this study, the number of independent variables is three at the most—natural frequency, damping ratio, and task duration in Equation (8)—demanding approximately 45 to 60 observations. The number of observations in this study was 9 observations per subject, with 22 subjects, totaling 198 and demonstrating the sufficiency of the subject number.

#### 2.3.3. Experiment Protocol 

Our experiments consisted of a pilot test and main experiments. The former was necessary to ascertain if the robot could exert *accurate* impedances—resistances, for simplicity—to the objects in contact. As those objects could be anything contactable, we used a desk surface instead of human subjects to that end. Hence the pilot test involved the robot alone, which contacts the surface by applying a specified resistance to the surface as it exerts reaction torque on the robot [51]. The main experiments involved both the robot and the subjects, with the robot exerting specified impedances and the subjects performing active extension-flexion motions against those impedances. Now the subjects are doing what the desk surface was doing.

For the pilot test, the parameters of the desired impedance used in the contact test were M_d_ = 1 kg·m^2^, B_d_ = 40 N·m·s/rad, and K_d_ = 16 N·m/rad. These parameter values resulted in a damping ratio, ζ = 5, creating overdamped characteristics to suppress overshoot during contact. The trajectory used was a fifth order polynomial trajectory with initial position = 0° at 0 s and end position = −90° at 4.5 s. Displacement was maintained for 5 s at the end position, and then we reversed the previous trajectory to return to the initial position. Two contacts in total were performed.

In the main experiments, we applied, through the robot, nine different desired impedances composed of the combination of three natural frequencies and three damping ratios (Table S28). These nine were sequenced in *random* order in order to make the stimuli completely *unpredictable* to the subjects so that we could examine how unpredictable stimuli affect brain activation. Further, as Table S29 illustrates, we offered different sequences of impedances to the 22 subjects; there was no sequence repeatedly applied to different subjects. 

The experiment adopted a block design that totals nine blocks, with each block consisting of a 30-s rest period and a 30-s task period (Figure S5a,b). The nine blocks accommodated the aforementioned nine impedances: in the task period of each block, while the robot exerted one of the nine different impedances, a subject strived to repeat active elbow extension-flexion motions, following the visual guidance, with three-second periods. Note, however, that the subjects were instructed to repeat nine times instead of ten, unless the task period spilled over to the rest period: no subjects could complete the tenth repetition in the task period, necessitating occupying time in the next rest period.

In the task period, the subject performed nine extension-flexion motions which were guided by visual guidance feedback. Figure S5c–e show the provided position, velocity, and acceleration trajectory to guide subject elbow motion. Note that we assigned the last 3 s of the fifth order trajectory as a transition period to the rest period for the reason mentioned in the last paragraph.

Rest duration is defined as the number of rest periods performed sequentially in Figure S5b, and task duration is also defined as the number of task periods performed sequentially in Figure S5b. 

### 2.4. Data Analysis

#### 2.4.1. Data Processing

Before calculating entropy, we used a bandpass filter based on the 500th-order finite impulse response for each channel data. In addition, a zero-phase digital filter was used to prevent phase shift. Since there is currently no consensus on the range of fNIRS filtering [62], we focused on the range of 0.01~0.34 Hz, which contains hemodynamics, harmonics of hemodynamics, and signals associated with our designed task. After filtering, linear detrending was performed for each channel. All processing was done using MathWorks’ Matlab 2017b.

#### 2.4.2. Entropy Calculation 

In order to evaluate entropy based on the measured optical density data, we adopted *sample entropy*, an algorithm developed to robustly estimate entropy on short and noisy data [63]. The value of embedding dimension(m) was 2 and the value was known as statistically useful for systems with slow dynamics [64,65]. The value of tolerance(r), according to [65], was 0.2× SD, with SD denoting the standard deviation of the data set. A function published in the Mathworks File Exchange site was used to calculate sample entropy [66]. When we estimated a sample entropy, we used a time series of optical density data for 30 s during either the rest period or task period. As a result, the calculated total number of entropy is 22 (the number of subjects) × 3 (the number of optical density signals) × 17 (the number of channels) × 18 (the number of periods) = 20,196.

#### 2.4.3. Signal Amplitude Calculation 

For comparisons of entropy with signal amplitude, we took a mean value for the *representative* signal amplitude. Just as we evaluated entropy based on 30-s time series data of rest and task periods, so we made its mean value represent its corresponding signal amplitude for 30 s. As a result, we calculated 20,196 mean values of the signal amplitude.

#### 2.4.4. Beta Value Calculation 

For the comparisons of entropy with beta value, we also calculated beta values. Beta values in principle were obtained by solving Y = X × beta + b, where Y is measured data, X is design matrix, and b is residue. To set a design matrix(X) we convolved the canonical hemodynamic response model and an array whose elements are all equal as one to set the design matrix. After setting the design matrix, we chose a method to estimate beta. In SPM, the default method to estimate beta is weighted least squares. Similar to signal amplitude, we calculated beta values from 30-s time series data, amounting to 20,196.

#### 2.4.5. Statistical Analysis

In order to investigate how the entropy computed in each channel is affected by various conditions, we employed linear mixed effect models. Also, for the comparisons of entropy with signal amplitude and beta value, we used exactly the same linear mixed effect models used to obtain statistical results for entropy, substituting only the entropy in the models with the signal amplitude or beta value. 

Since the conditions, such as rest and task states, task duration, or task strength—this consists of natural frequency and damping ratio—can be controlled, we set them as independent variables. In contrast, however, we set the subjects to random effects to take into account the variability of each subject: too much individual difference and uncontrollable factors (e.g., gender, mood, condition, etc.) Unless random effect is not included in the model, the effect of the dependent variable cannot be found. Each of the models we employed is composed of Wilkinson-Rogers notation [67], that is, a description of the conceptual relationship between a dependent variable to independent variables, and algebraic notation as follows:

The model to check the difference between the entropy of the rest and task states was formulated as the following:

(4) Entropy ~ State + (1|subject) ,

(5) $$y_{\mathrm{wjkm}}=\;\beta_{0,\mathrm{wj}}+\;\gamma_{\mathrm{wjm}}+\beta_{\mathrm{s,wj}}\cdot {\mathrm{State}}_{k}\;+\varepsilon_{\mathrm{wjkm}},$$

where y: Sample entropy, $\beta_{0}{,\beta }_{s}$: Intercept, state slope, γ: Random intercept, ε: Residue, w = 1, 2, 3 (wavelength: 780, 805, and 830 nm), j = 1, 2, ⋯, 17 (Channel), k = 1, 2, 3, ⋯, 18 (odd = rest period, even = task period), m = 1, 2, ⋯, 22 (subject).

The model was made to check the relationship between rest duration and entropy in the rest state as follows:

(6) Entropy ~ Rest duration + (1|subject) ,

(7) $$y_{\mathrm{wjkm}}=\;\beta_{0,\mathrm{wj}}+\;\gamma_{\mathrm{wjm}}+\beta_{\mathrm{t,wj}}\cdot \mathrm{Rest}\;{\mathrm{duration}}_{k}\;+\varepsilon_{\mathrm{wjkm}},$$

where y: Sample entropy, $\beta_{0},\;\beta_{s}$: Intercept, rest duration slope, γ: Random intercept, ε: Residue, w = 1, 2, 3 (wavelength: 780, 805, and 830 nm), j = 1, 2, ⋯, 17 (Channel), k = 1, 3, 5, ⋯, 17 (odd = rest period), m = 1, 2, ⋯, 22 (subject).

The following model was set up to investigate the relationship of entropy with each of three independent variables (task duration, natural frequency, damping ratio):

(8) $$\mathrm{Entropy}\;\sim \;\mathrm{Task}\;\mathrm{duration}+\;\omega_{n}+\zeta +\;(1|\mathrm{subject})\;,$$

(9) $$y_{\mathrm{wjkm}}=\;\beta_{0,\mathrm{wj}}+\gamma_{\mathrm{wjm}}+\beta_{\mathrm{t,wj}}\cdot \mathrm{Task}{\mathrm{duration}}_{k}+\beta_{\mathrm{n,wj}}\cdot \omega_{\mathrm{n,km},}\;+\beta_{\mathrm{d,wj}}\cdot \zeta_{\mathrm{km}}\;+\varepsilon_{\mathrm{wjkm}},$$

where y: Sample entropy, $\omega_{n}\;$: Natural frequency, ζ: Damping ratio, $\beta_{0},\;\beta_{t},\;\beta_{n},\;\beta_{d}$: Intercept, slopes of task duration, natural frequency, and damping ratio, γ: Random intercept, ε: Residue, w = 1, 2, 3 (wavelength: 780, 805, and 830 nm), j = 1, 2, ⋯, 17 (Channel), k = 2, 4, 6, ⋯, 18 (even = task period), m = 1, 2, ⋯, 22 (subject).

The fitting of the model used maximum likelihood, and the two-side t-test was used for statistical analysis of fixed effect coefficients. All calculations were done using Mathworks’ Matlab 2017b.

#### 2.4.6. Brain Activation Map

In order to investigate how the channels identified by the entropy difference between the rest state and task state consisted with previous brain activation mapping results, we constructed activation maps based on oxyhemoglobin data using NIRS SPM [11]. To that end, we first conducted an individual analysis for each subject according to the procedure in [45] and then collected the results for group analysis to make the activation maps. 

In those analyses, we strived to reduce family-wise errors by making the *p*-value correction with the expected Euler characteristic approach, based on Lipschitz-Killing curvature (LKC). Differing from the individual analysis, the group analysis opted for the *default* setting of NIRS-SPM. All calculations associated with both analyses were carried out by using Matlab 2012b.

## 3. Results

After we verified the accurate generation of the mechanical impedance that modulates the stimulus to the brain during task execution, we examined the entropy difference between the rest state and the task state to see if entropy can discern brain activation changes between the upper limit and lower limit of the stimuli. The in-between stimuli were applied by varying task intensity in terms of task duration and task strength, embodied by mechanical impedance, in order to inspect their effects on brain activation. In addition, similarities and differences were identified by comparing signal amplitude and beta evaluation of brain activation with the entropy evaluation of brain activation. To confirm the validity of the location identified by entropy, we crosschecked the identified location with the brain activation map obtained from NIRS-SPM. 

### 3.1. Control Verification

#### 3.1.1. Validation of Impedance Control

**Figure 1:**
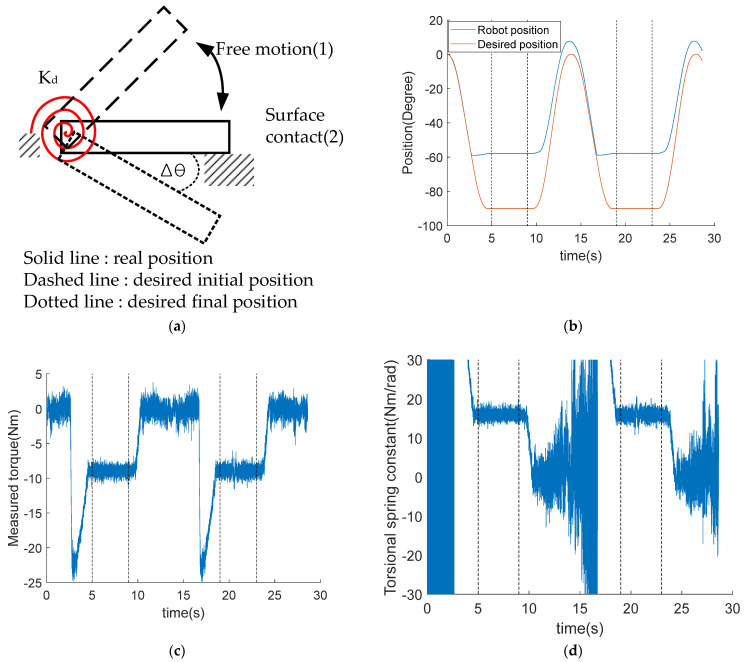
Contact test to validate mechanical impedance control: (**a**) Schematic of contact test for checking desired spring constant. This experiment consisted mainly of two parts, free motion and surface contact notated by (1) and (2) respectively. During free motion, the robot approached the stiff surface. After contact with the stiff surface, the robot remained in its position because the robot cannot pass through a surface. This situation makes a difference, Δθ, between real position and desired position. Using the difference and measured torque, we estimated spring constant; (**b**) Desired and actual position trajectories. red solid line is desired trajectory and blue line is actual trajectory robot moved. Vertical broken lines are used to distinguish surface contact from free motion. Due to contact, the robot did not follow the desired trajectory and gave a difference (Δθ) between desired and real position. An error of approximately 30 degrees occurred in steady state intervals; (**c**) Measured torque when robot operated. There are three phases in this test. first is free motion before contact. In this phase, there is no reaction force theatrically. Second is impact phase. There is abrupt change of reaction torque. The last is steady phase, where the robot stays in a specific position. If the robot is fixed perfectly, the robot will stay at the contact position. However, in our case, the robot was not fastened perfectly, it moved slightly; (**d**) Estimated torsional spring constant over time. Estimation was valid only between steady state because simplified estimation was used. The biggest reason why a simplified estimation was used was that acceleration and velocity were quite noisy because of numerical differentiation.

As a result of the preference to realize differentiated stimuli, we considered it important to accurately impose the desired mechanical impedance on the subject, when the exoskeletal robot was in contact with him or her. For this purpose, we first performed a contact test where the robot was made to touch a surface (see Figure 1a), not a human subject, and examined if the robot achieved the desired spring constant, the simplest form of the desired impedance. Then, we made the impedance measurement, while human subjects wearing the robot made extension-flexion motions against the impedance created and posed by the robot.

##### Contact Test

After having the robot tip touch a desk surface, we measured the joint torque and joint position, from which we calculated the achieved spring constant (Figure 1a). The desired impedance parameters were selected as M_d_ = 1 kg·m^2^, B_d_ = 40 N·m·s/rad, and K_d_ = 16 N·m/rad. 

Figure 1b displays the desired position (red line) of the robot and the actual position of the robot (blue line) that oscillated from the free motion state to the contact state as is shown in Figure 1a. The contact state, after the slight transient responses due to the impact at 3 s and 17 s, converged to a fixed position at approximately −60°. The measured torques during those oscillations are shown in Figure 1c. In the free motion state, the torque value remained 0 owing to no resistance torque before the contact state started at 3 s and 17 s, involving sharp peak torques, due to the impact. The peak converged to a constant value of 8.3776 Nm in both periods of 5~9 s and 19~23 s, when the joint angle converged to −60°. 

Since the acceleration and velocity were virtually nonexistent, Equation (2) in Method leads to ${\hat{K}}_{d}\;=\;\tau_{measure}\;/\Delta \theta$. In Figure 1d, ${\hat{K}}_{d}$ is plotted, and their mean and standard deviation were calculated as 15.9996 and 1.0045 Nm/rad in 5~9 s, and 15.9909 and 1.2067 Nm/rad in 19~23 s, respectively. This value showed that the desired spring constant, K_d_ = 16 N·m/rad, was implemented very closely.

##### Impedance Error Measurement

Based on the above spring constant verification, when subjects do elbow extension-flexion motions while wearing the robot, the impedance error, in terms of the discrepancy between the desired impedance and the actually exerted one, was evaluated to confirm that the robot accurately generated the desired impedance. Each of the 22 subjects performed the task according to the block design paradigm, which consisted of nine rest-task blocks, with each block comprising a 30 s rest period and a 30 s task period, with each task repeating nine extension-flexion motions. For the nine task periods, each subject was applied in a random order with nine different desired impedances coming from different combinations of the natural frequency and damping ratio. To evaluate the net impedance error, we used Equation (3) (in Methods).

**Figure 2:**
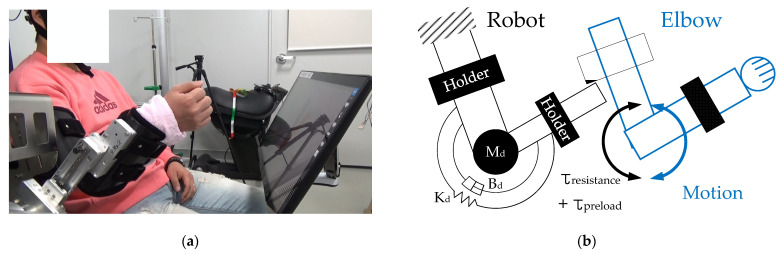

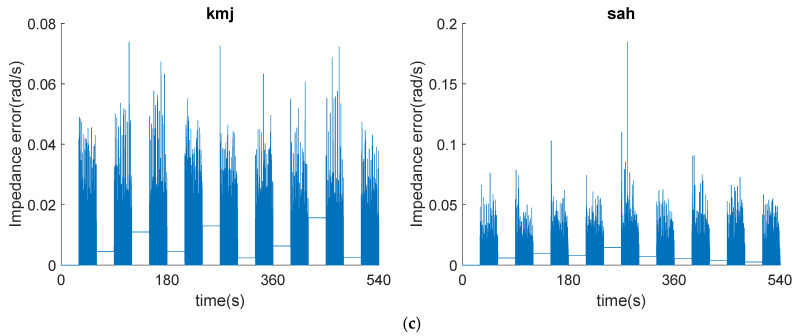
A photo of subject wearing exoskeleton robot, schematic of mechanical impedance against elbow joint, and subjects’ graphs of Impedance error: (**a**) A subject wearing exoskeleton robot conducted elbow extension-flexion motions. Exoskeleton robot provided resistance torque based on desired mechanical impedance and preload to subjects (Video S1); (**b**) To apply resistance torque and preload to elbow, mechanical impedance was introduced and preload was introduced into desired mechanical impedance as constant. Desired Inertia, damper, and spring component make a resistance torque that is proportional to acceleration, velocity, and displacement by elbow motion (extension-flexion motions); (**c**) To check the degree of accuracy of desired mechanical impedance, impedance errors of subjects were calculated using Equation (3). Two subjects’ results are illustrated here. Spikes occurred when the subject forearm collided with the upper arm.

Figure 2a shows the subject wearing the robot, while Figure 2b illustrates how the robot is exerting the resistance torque corresponding to the desired impedance (M_d_, B_d_, and K_d_) and the preload to the elbow joint when the subject performs active movements. Figure 2c shows typical impedance errors for two subjects calculated with Equation (3)—note that they are bound. In fact, the impedance error for the 22 subjects had an average value of 0.0061 rad/s and a standard deviation of 0.0056 rad/s.

### 3.2. Entropy Changes Owing to Physical Stimuli

#### 3.2.1. Entropy Difference between Rest and Task

In our investigation, if there were any noticeable entropy differences between the rest state and the task state, we observed that the difference in entropy between the two states was significant and that the entropy of the task state had larger values than that of the rest state. To elaborate, we calculated the entropies of the 22 subjects based on the optical density data, generated with three wavelengths, measured at 17 channels for nine rest periods and nine task periods, respectively. Note that the data were acquired while each subject was performing the extension-flexion motions under different desired impedances according to the procedure described in the impedance error measurement. The data were fit to the linear mixed model in Equation (4) in Methods to produce the essential parameter values at the 17 channels, such as the slope, intercepts, along with the *p*-values and the confidence intervals (CI). These values are displayed according to the wavelengths in Tables S1–S3.

**Figure 3:**
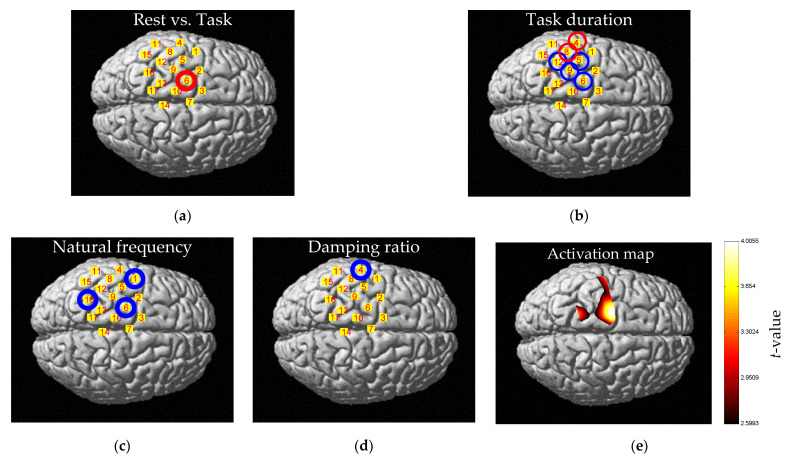
Significant Channels, brain activation map, and fitting results: (**a**) Statistically significant channel of difference between the rest and task: CH6. A red circle means statistical results are significant in all wavelength data. Details are in Tables S1–S3; (**b**) Statistically significant channel of change of entropy over task duration: CH4, 5, 6, 8, 9, and 12. A red circle means statistical results are significant in all wavelength data. A blue circle means statistical results are significant in two wavelength data. Details are in Tables S7–S9; (**c**) Statistically significant channel of change of entropy by natural frequency: CH1, 6, and 16. A blue circle means statistical results are significant in two wavelength data. Details are in Tables S7–S9; (**d**) Statistically significant channel of change of entropy by damping ratio: CH4. A blue circle means statistical results are significant in two wavelength data. Details are in Tables S7–S9; (**e**) Brain activation map calculated by NIRS-SPM. One-sided *t*-test was used to find the activated area, and the threshold *p*-value was 0.05. To compensate family-wise error, expected Euler characteristic approach based on Lipschitz-Killing curvature (LKC) was used.

Close inspection of the *p*-values and the CIs in the three tables reveals that there is one channel that consistently showed a significant difference in the entropies between the rest and the task. That channel is CH6, the location of which is marked with a red circle in the standard brain map in Figure 3a. At CH6, as the three tables display, the *p*-values are less than 0.05 and the CIs have nonzero values in all three wavelengths. In addition to establishing that the difference is significant between the two states, we can compare the entropies of the two by considering the effect sizes (slope) and the intercepts. Specifically, the three tables list that the effect sizes at CH6 in 780, 805, and 830 nm are 0.0048, 0.0029, and 0.0024, respectively, and the intercepts in these wavelengths are 0.0367, 0.0357, and 0.0307, respectively. These data immediately lead to the conclusion that the entropy at the task state is larger than the rest state by 12.97% in 780 nm data, 8.02% in 805 nm data, and 7.91% in 830 nm data.

#### 3.2.2. Entropy Change over Rest Duration

In addition to establishing the rest–task entropy difference, we examined whether entropy was subject to any change during the rest state. If it was, that meant that there could have been a *carry-over effect* from the previous task state or that the rest state could be affected by unidentified or unknown factors, and that the result as to the rest–task entropy difference could be inconclusive or even erroneous. In order to investigate the change in the rest state, we applied the linear mixed model to obtain Equation (6) in Methods. To $y_{wjkm}$ in this equation, we substituted the entropy data pertaining to the rest state, out of all the entropy data obtained from all subjects, for all periods of rest and task, from all channels, and in all wavelengths. As a result, we obtained the values of β_0_ and β_t_, the intercept and the slope, along with the other parameters, which are listed in Tables S4–S6. 

Having observed the CI (not including 0) and *p*-value (<0.05) of the fixed effect, we found CH7 in 830 nm displayed a significant entropy change at rest duration. Since significance was found in only one channel out of the 17 and only one wavelength out of the three, effectively one case out of 51, we concluded that there is no significant entropy change at the rest state in our problem.

#### 3.2.3. Relationship between Task Duration and Entropy

To investigate the relationship between task duration and entropy, we used Equation (8) (Methods) and obtained the results in Tables S7–S9. 

A close observation exposed that there were two channels whose task duration had a significant relationship with the entropy at all three wavelengths, and four channels that showed such significant relationship at two wavelengths. These were CH4 and CH8 which showed such relationship at all three wavelengths and these were CH5, CH6, CH9, and CH12 which showed such relationship at 780, 830 nm. The two channels that showed such significant relationship at all three wavelengths were marked by red circles and the four channels that showed such significant relationship at two wavelengths were marked by blue circles at the standard brain map in Figure 3b.

**Figure 4:**
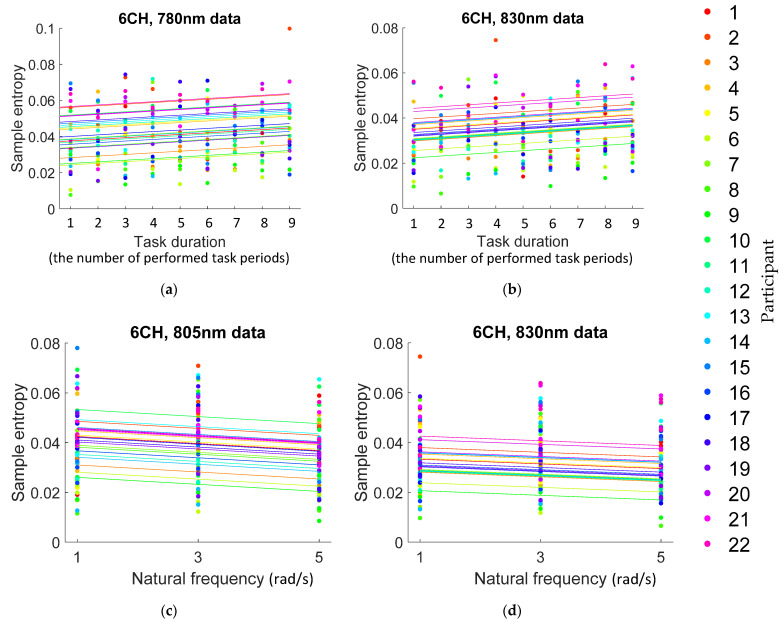
Fitting results at CH6: (**a**,**b**) Fitting results of statistically significant channel of change of entropy over task duration. Figure 4a,b were plotted using wavelength 780, and 830 nm data respectively. Each subject was distinguished by assigned colors. Details are in the article and Tables S7–S9; (**c**,**d**) Fitting results of the statistically significant channel of change of entropy by natural frequency. Figure 4c,d were plotted using wavelength 805, and 830 nm data respectively. Each subject was distinguished by assigned colors. Details are in the article and Tables S7–S9.

Of the six, we chose to focus on CH6 only, because it was noted as the only channel with a significant entropy difference between the rest and the task. According to Tables S7 and S9, CH6 had positive effect sizes at 780 and 830 nm, and its corresponding intercepts were 0.0408 and 0.0334, respectively, signifying that the entropy increased as the task duration increased. The increase rates are 2.19%, 2.38%, respectively. This rate of increase is shown by the slopes of the straight line group in Figure 4a,b; each individual has his or her own color—A close inspection of Figure 4a,b shows that the interval was two between task duration = 1 and task duration = 2 so that the increase rates in entropy between task duration = 1 and task duration = 2 were 2.19%, 2.38% at wavelength data 780 and 830 nm, respectively. Comparing task duration = 1 and task duration = 9, the increase rates in entropy from task duration = 1 and task duration = 9 were 17.48%, 19.01% at wavelength data 780 and 830 nm, respectively.

#### 3.2.4. Relationship between Task Strength and Entropy

As was mentioned in the Introduction, mechanical impedance can be characterized by natural frequency (*ω*_n_) and damping ratio (ζ), so we investigated the relationship between these two parameters and entropy. To this end, we used Equation (8) (Methods) to obtain the results in Tables S7–S9. 

A close observation exposed that there was no channel at all whose natural frequency had a significant relationship with the entropy at all three wavelengths, but several channels showed such significant relationship at two wavelengths. These were CH1, which showed such relationship at 780, 805 nm; CH6 at 805, 830 nm; and CH16 at 780 nm, 830 nm, respectively. The three channels are marked by blue circles on the standard brain map in Figure 3c. 

Of the three, we chose to focus on CH6 only, because it was noted as the only channel with a significant entropy difference between the rest and task. According to Tables S8 and S9, CH6 had negative effect sizes at 805 and 830 nm, and its corresponding intercepts were 0.0420 and 0.0334, respectively, signifying that entropy reduced as the natural frequency increased. The reduction rates were 3.37% and 2.80%, respectively. This rate of decline is shown by the slopes of the straight line group in Figure 4c,d; each individual has his or her own color. A close inspection of Figure 4c,d shows that the interval was two between *ω*_n_ = 1 and *ω*_n_ = 3, so that the reduction rates in entropy from *ω*_n_ = 1 to *ω*_n_ = 3 are 6.73% and 5.60% at wavelength data 805 and 830 nm, respectively. Comparing *ω*_n_ = 1 and *ω*_n_ = 5, the reduction rates in entropy from *ω*_n_ = 1 to *ω*_n_ = 5 are 13.47% and 11.19% at wavelength data 805 and 830 nm, respectively.

The results of the relationship between damping ratio and entropy are shown in Tables S7–S9, and Figure 3d. Like the natural frequency case, there was no significant channel in all three of the wavelength data and there was only one channel with significant fixed effect in the two of the wavelength data, which was CH4. However, since CH4 is not a location where significant entropy differences were found between rest and task, only CH6 was, no additional interpretation was attempted. 

### 3.3. Comparision of Entropy Change with Signal Amplitude and Beta Value

Signal amplitude and beta value have been widely used to evaluate brain activation, so we calculated signal amplitude and beta value of brain activation and conducted statistical analyses using both indicators to compare with entropy of brain activation (Tables S10–S18 for signal amplitude, Tables S19–S27 for beta value). It was found that the signal amplitude and the beta value of the task state were greater than those of the rest state in whole channels, except CH8 and CH12, and that the signal amplitude and the beta value increased with increasing task strength in whole channels. All results were summarized in Table 1 and Table 2 in order to check commonalities and differences with entropy.

### 3.4. Comparision of Entropy Change with Brain Activation Map

When performing motion for a given desired impedance, group analysis mapping (Figure 3e) was performed using NIRS-SPM to check the relationship between the locations of the channels found in the previous results and the conventional activation map. Looking at the group analysis mapping result, it was confirmed that the activated channels were similar to the channels in Figure 3c, that is, the channels had a statistically significant relationship obtained from the relationship between *ω*_n_ and entropy. In particular, it was confirmed that CH6 has a large *t*-value, and, for the remaining locations, it was also confirmed that activation was found in a location similar to the location shown in Figure 3c.

## 4. Discussion

### 4.1. Desired Impedance Is Achieved

The result in Figure 1d shows that spring constant, or stiffness, the simplest form of impedance, could be accurately achieved as desired, supporting the feasibility of accurate production of a complete impedance. The impedance error of 22 subjects, the error between the desired and the achieved, is sufficiently small (0.0061 rad/s) for the desired impedance. That means we could accurately create impedance, both in total and in proportion, as an arbitrary combination of the natural frequency and the damping ratio. That also means we could accurately produce and differentiate the stimuli (or the dose) in total and in proportion. This impedance was applied to all subjects in the subsequent experiments. The data in Figure 2c shows the impedances actually used to investigate brain activation. Besides the desired impedance being determined as a combination of arbitrarily selected natural frequency and damping ratio, these two were used as independent variables for statistical analysis.

It is noteworthy that the smallness of the impedance error is not a sufficient condition, but a necessary one, for a perfect realization of desired impedance. Nevertheless, it is a good indication of accuracy. This accuracy, widely recognized as difficult to achieve with conventional robots, could have been achieved thanks to the effectiveness of the control technique introduced in Section 2.2.2.

### 4.2. Brain Activation Could Be Evaluated by the Entropy

In this subsection, we endeavor to establish entropy as a measure of the quantity of brain activation in response to physical stimuli. To this end, we are going to discuss the bottom-line question as to whether the presence of physical stimuli (tasks) makes any relevant difference in entropy from their absence (rest). Then, we will discuss how entropy varies as stimuli intensify varies in terms of task duration and task strength.

#### 4.2.1. Rest State and Task State

The results in Tables S1–S3, and Figure 3a show that entropy in the task state is greater than that in the rest state and that there is no significant entropy change at the rest state in CH6. These results confirm a previous finding that entropy is greater at a task than it is at rest [39], which was estimated from event-related fMRI time series. Moreover, the results in Tables S4–S6 show that entropy at the rest state does not change, so the phenomenon that entropy is greater at the task state than it is at the rest state is not by chance but due to the task.

Apart from monitoring entropy changes, some research works [19,68,69] have observed change of brain activation per se. Those studies reported that brain activation increased as stimuli shifted from rest to task. It is noteworthy that one of them invasively examined the brain activation of monkeys and found that neural activity increased when a visual stimulus was given and that it was maintained only while the visual stimulus was present [19].

In summary, as brain activation increases upon the introduction of stimuli from their absence, so does entropy, qualifying itself as a relevant measure for brain activation change from rest to task.

#### 4.2.2. Task Duration

The results in Tables S7–S9, along with Figure 4a,b, show that entropy increases as task duration increases. In our experiment, task duration could mean the numbers of the extension-flexion motions or, more broadly, the number of stimuli applications. Since nine different impedances were randomly provided to each subject, the extension-flexion motions under each impedance were completely unpredictable and unfamiliar to him or her, posing unfamiliar stimuli. To rephrase our finding, therefore, entropy increases as an unfamiliar task is prolonged, or, more generally, it increases with prolongation of unfamiliar stimuli. 

As to the effect of task duration on brain activation, a previous review paper [70] summarized that brain activation generally increases with an increased number of stimulus applications when it is unfamiliar, and that the activation decreases with a familiar stimulus, even when its applications are prolonged. A finding in agreement with that has been reported in that brain activation, a value assessed by signal amplitude, increased when an unfamiliar stimulus was provided [71]. Similarly, in an experiment introducing low-visible pictures as a stimulus (an unfamiliar stimulus), brain activation initially increased and then diminished with the number of repetitions [72], forming an inverted U-shape curve. 

The agreement of brain activation with entropy in the presence of prolonged unfamiliar stimuli supports entropy as a qualified measure for brain activation.

#### 4.2.3. Task Strength

The results in Tables S7–S9, and Figure 4c,d indicate that entropy decreases as task strength increases, a somewhat counterintuitive result. However, this phenomenon has already been observed in previous research: the entropy of EEG signal decreases as the difficulty level of an arithmetic calculation increases [44]; and the entropy of neural model output decreases as the intensity of input related to task strength increases in the neural model simulation [73].

How about the brain activation in this case? Does brain activation also decrease as task strength increases? In fact, a previous study has reported that, depending on the torque direction [74], flexion torque or extension torque, brain activation actually decreased as its magnitude increased. However, this result is only a part of far more complex and intricate phenomena, which we are going to discuss a little more in depth owing to its close connection to our study.

In the study [74], through invasive measurements of electrical signals from 160 neurons in the precentral gyrus, they found that neural activities either increased or decreased, depending on the direction and magnitude of each joint torque, as monkeys exerted joint torques at both the elbow and shoulder. To elaborate, of the 68 neurons (out of 160) that responded to the elbow motion, half of them (call it Group A, as in Table S30) displayed reduced discharge rate, or reduced brain activation, upon the monkeys’ exertion of elbow extension torque, while the other half (Group B) showed increased discharge rate upon the same torque. The elbow flexion torque, on the other hand, involved both increased discharge rate of Group A and decreased rate of Group B. 

As Table S30 illustrates, the increase and decrease of brain activation are determined not only by the magnitude of exerted torque but also by its direction, and by two co-existent neuron groups alternating between its increase and decrease opposite to each other. These two directions and two groups create four statuses of brain activation denoted by Cases 1–4 in the table. 

Those two neuron groups are responsible for the activation/deactivation of four muscles, two muscles dedicated to extension m1, m2, and the other two to flexion m3 and m4. Specifically, the extension torque requires the activation of m1 and m2, which in turn increases the discharge rate of Group B, while deactivating m3 and m4, decreasing that of Group A in the end. For the flexion, on the other hand, m3 and m4 need to be activated with m1 and m2 being deactivated, resulting in both increased discharge rate of Group A and decreased one of Group B [74]. 

Of those four cases, Case 1 has been predicted by our entropy result, while Case 2 was simply unobservable and Cases 3 and 4 were unavailable because our experiment involved exertion of extension torque only. 

The fact that the exertion of torque, be it extension or flexion, accompanies decrease of neural activation is counterintuitive and at the same time demonstrates the intricacy and complicated nature of brain activation, which previous studies, including signal amplitude, have been unable to discern. 

Although it is unknown whether entropy could express brain activation completely induced by direction of torque (flexion or extension) and task strength, entropy at least partially explains the increase or decrease in brain activation. Signal amplitude, which has been recently popular, increases as the size of task strength itself increases, regardless of the direction of task strength [75], so it does not represent the complex phenomenon related to the increase or decrease of brain activation according to the direction of task strength. In conclusion, the phenomenon of decreased brain activation cannot be explained by signal amplitude, and, using entropy, a delicate and counter-intuitive phenomenon can be explained. Future study is needed to determine how entropy was able to capture this phenomenon and whether it is possible to capture another case.

### 4.3. Comparison with Signal Amplitude and Beta Value

In comparison with signal amplitude and beta value, we have found that entropy has two advantages. First, entropy has the potential to encapsulate richer information than signal amplitude and beta value. As Table 1 shows, entropy measures brain activation due to both task strength and task duration, while signal amplitude and beta value measure the effect of task strength only. This means entropy encompasses information not only about how strongly the task was being performed but also about how many times it was repeated. 

Second, entropy change appears in a *localized* fashion, conforming to the *modularity theory* [76], but signal amplitude and beta value change do not. As Table 2 demonstrates, the former is restricted to some channels, whereas the latter is widespread at all channels, except for CH8 and CH12, contradicting the theory which states that a specific brain area, being responsible for a specific body function, is activated by that function [76]. Although there are functions that cannot be explained by modularity theory, at least sensory and motor functions are known to be well explained by modularity [77]. In other words, if a specific motor function is performed, activation should be found only in the corresponding brain area. Signal amplitude and beta value change, however, were observed at almost all channels, failing to indicate modularity of brain activation. In contrast, entropy change appears only in specific regions related to elbow motion, demonstrating a better indication of brain activation. 

Compared to the signal amplitude approach and the beta value approach, using entropy has the downside of additional computation cost, as it is obtained by processing time series data of signal amplitude. In our study, we used time series data for 30 s to compute one entropy point, consuming 0.0787 s, on average, of computation time based on Xeon E5–2620. The computation of the whole entropy data used in this article, 20,196 entropy data, took 1590.7258 s, less than 30 min. That is the additional computation cost required. Note that the computations of 20,196 signal amplitudes and 20,196 beta values took 0.8078 s and 2.7905 s, respectively.

Therefore, it can be advantageous to use entropy when measuring brain activation because it can yield unidentified and rigorous information compared to signal amplitude and beta value.

### 4.4. Physiological Interpretations

In the previous discussion, CH6 was noted for being the only channel that exhibits a statistically significant entropy difference between the rest and task. In addition, it is the only channel marking such differences in response to both task duration and task strength, as is shown in Figure 3a–c. In fact, this channel, located at the primary motor cortex area, is known to be related to elbow function [78]. Moreover, the position of CH6 was identified by the widely used brain activation map which was used to find some physiological location relating to a task (Figure 3e). The fact that entropy is capable of identifying such a physiologically important location is significant, further supporting its capability as an index for brain activation.

Besides, the three channels in Figure 3c that mark a significant entropy change in response to task strength are located at the primary motor cortex (CH6), the parietal cortex (CH16), and the premotor cortex (CH1), respectively. These channels could be interpreted as forming a cortical network used for goal-directed movement [79,80]. Specifically, the parietal cortex (CH16) converts visual information into motor commands and transmits them to the premotor cortex (CH1) [81], which generates a specific motor command to be executed at the primary motor cortex (CH6) [82].

Of the six channels in Figure 3b that displayed significant responses to the task duration, only CH6 is related to elbow, while the rest (CH4, 5, 8, 9, 12) relate to other parts, such as wrist, hand, face, etc. [78], demanding explanation. It appears the movement was not completely controlled (Figure 3b,d). Fortunately, however, since those channels exhibited no significant entropy difference between the rest and task, they could be excluded from brain activation investigations.

### 4.5. Reason for Not Considering Damping Ratio

When investigating the effect of the task strength on brain activation, we did not consider damping ratio because it did not display any relationship with the entropy at CH6 (Figure 3d), the only channel where significant difference in entropy was observed between the rest and task. Having confirmed that entropy nevertheless could express brain activation, we may deduce that damping ratio in effect had little contribution to brain activation. 

Since the damping ratio is an important component of task strength, its lack of contribution demands explanation. A close inspection of our experiments offers a clue. In our experiments, while a visual guide was provided to prompt subjects to track a sequence of positions, so that natural frequency (or spring constant) could be constantly perceived, no such guide was offered for velocity, which is directly related to damping ratio. The lack of such guide could make velocity completely arbitrary to each subject at each moment, making it difficult for damping ratio to actively participate in brain activation and thereby contributing natural frequency to become the dominant component of the task strength. Then the brain could have activated in response to natural frequency only by selective perception [83,84]. Further investigation should include visual guides for velocity, too, in order to induce the active participation of damping ratio.

### 4.6. Consideration of Entropy Calculation 

It is known that the stationarity of the signal is required as a prerequisite for entropy utilization, and if this is not satisfied, entropy is calculated incorrectly [85]. After checking stationarity, we found that our signal was nonstationary. However, the linear detrending we performed is one of the methods to minimize nonstationary shifts in mean [86]. In the wide-sense stationarity test, 1/3 of our data had stationarity [87].

Also, it has been reported that various entropy calculation methods showed different results depending on the methods [88], and using a local version of sample entropy can find the nonlinear features of the signal more robustly than the traditional sample entropy definition [89]. In future brain activation studies, a method that compensates for signal non-stationarity should be applied. It is also necessary to compare the results by using various entropy calculation methods and improved sample entropy.

### 4.7. Summary

It was confirmed through experiments and previous studies that brain activation could be expressed using entropy. In addition, it was possible to confirm that the use of entropy has superiority in comparison with signal amplitude beta value that are widely used, and the practical application of entropy was also confirmed by confirming that the regions identified using entropy have physiological meaning. Although there are phenomena that cannot be clearly explained and unconfirmed results, entropy can be a useful measure when studying the intensity of the brain’s response to a given task.

## Figures and Tables

**Table 1:** Comparisons among the tendencies of the relationships of entropy, signal amplitude and beta value.

Relationship	Entropy	Signal Amplitude	Beta Value
Rest vs. Task	Entropy (Rest) < Entropy (Task)	Signal amplitude (Rest) < Signal amplitude (Task)	Beta value (Rest) < Beta value (Task)
Rest duration	ns ^1^	ns ^1^	ns ^1^
Task duration (TD)	TD ∝ Entropy	ns ^1^	ns ^1^
Natural frequency (NF)	NF ∝ 1/Entropy	NF ∝ Signal amplitude	NF ∝ Beta value
Damping ratio (DR)	ns ^1^	ns ^1^	ns ^1^

^1^ ns: not significant.

**Table 2:** Comparisons among the significant channels of the relationships of entropy, signal amplitude and beta value.

Relationship	Entropy	Signal Amplitude	Beta Value
Rest vs. Task	CH6	Whole CHs. except CH8, 12	Whole CHs. except CH8, 12
Rest duration	ns ^1^	ns ^1^	ns ^1^
Task duration (TD)	CH4, 5, 6, 8, 9, 12	ns ^1^	ns ^1^
Natural frequency (NF)	CH1, 6, 16	Whole CHs.	Whole CHs.
Damping ratio (DR)	CH4	ns ^1^	ns ^1^

^1^ ns: not significant.

## Data Availability

The data that support the findings of this study are available from the corresponding author upon reasonable request. The data are not publicly available due to privacy.

## Supplementary Materials

The following supporting information can be downloaded at: https://www.mdpi.com/article/10.3390/e24040556/s1, Figures S1–S5; Tables S1–S30; Video S1.
