# Supplementary material for: Entropy Could Quantify Brain Activation Induced by Mechanical Impedance-Restrained Active Arm Motion: A Functional NIRS Study
Source: Entropy (Basel). 2022 Apr 15;24(4):556. doi: 10.3390/e24040556 (PMC9024511; doi:10.3390/e24040556)
Supplement: Supplementary file 1 [file entropy-24-00556-s001.zip › Figure_S.pdf]

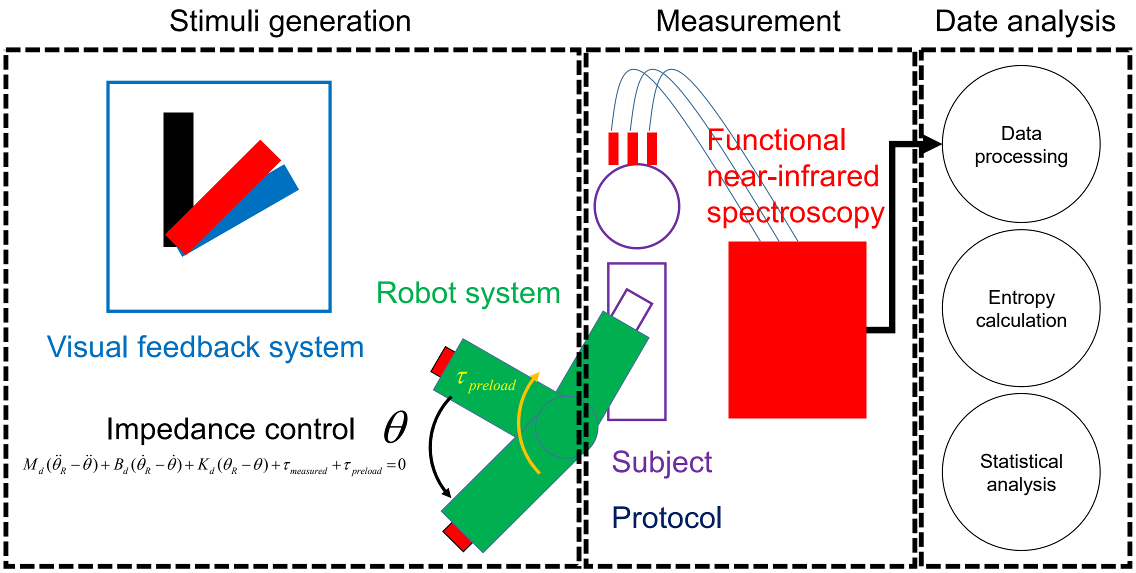

Figure S1. Methods configuration schematic: Methods configuration consists of stimuli generation, measurement, and data analysis. Each part has subparts. Put simply, we make stimuli by using the robot with impedance control and visual feedback to the subject. While providing stimuli, we measured brain activation from each subject by using functional near-infrared spectroscopy based on experiment protocol. After that, we conducted data analysis such as data processing, entropy calculation, statistical analysis, etc.

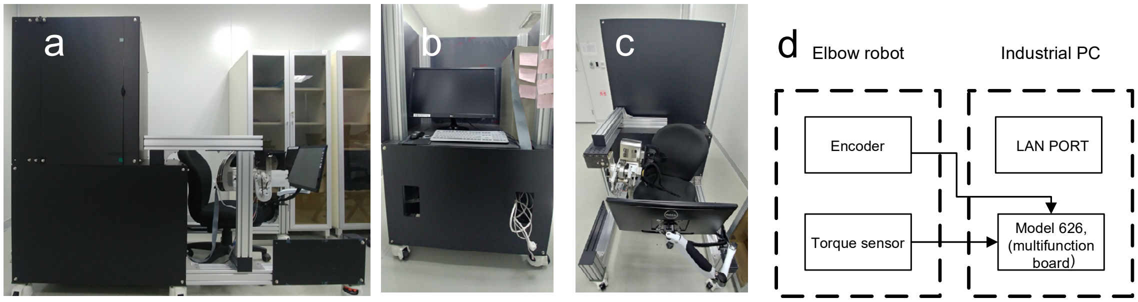

Figure S2. Robot system: (a, b, c) Packaged robot system pictures. (a) is a side view of the packaged robot system, (b) is a rear view of the packaged robot system, where is the operator's workspace, and (c) is a front view of the packaged robot system, where is the subject's workspace; (d) Robot and industrial PC connections. Robot system provides position data from encoder and measured torque data from torque sensor. Position data is digital signals of two channel and torque data is an analog signal.

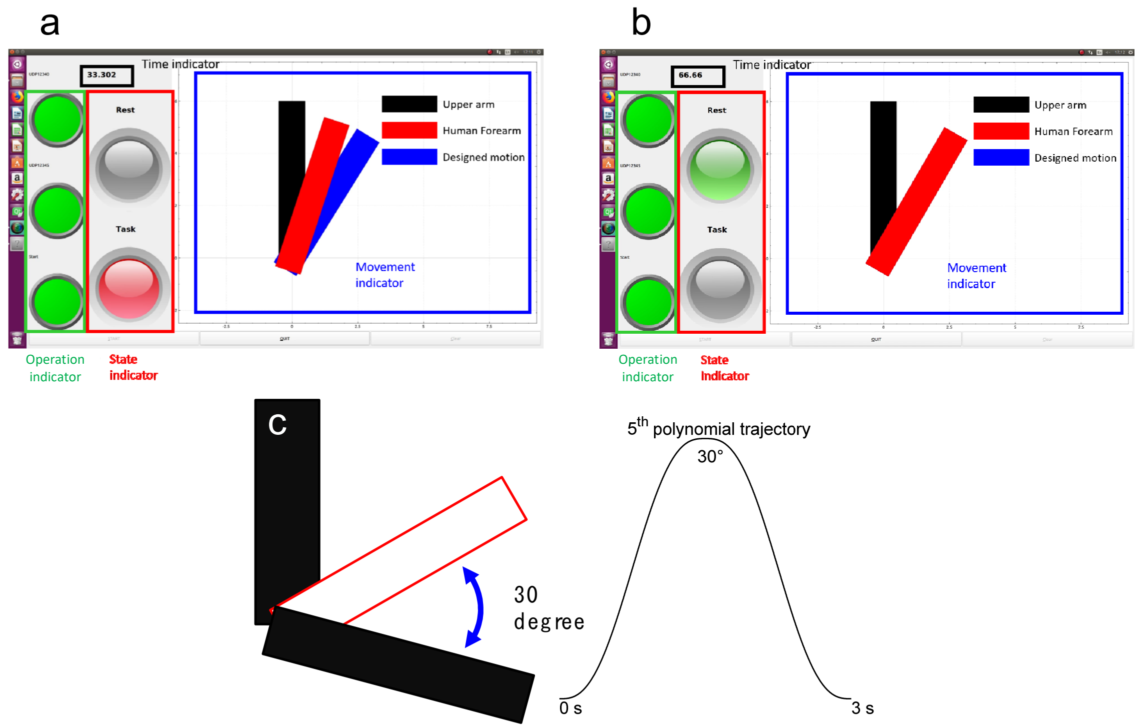

Figure S3. Visual feedback system: (a, b) Visual feedback graphical user interface (GUI). Visual feedback GUI structure consists of operation, state, time, and movement indicators. Operation indicator shows states of fNIRS and robot. If communication works, green LED lights are on. State indicator tells participants which period you are in. (a) is the task period and (b) is the rest period. Time indicator tells subjects elapsed time. Movement indicator is used to provide reference trajectory and participant's current position. Red rectangular represents the subject's forearm. Blue rectangular represents virtual arm which follows reference trajectory. Black rectangular is used to provide intuition, which this figure means upper arm. (c) Provided guide motion through visual feedback. Guide motion was designed by 5th polynomial with 30° amplitude and 3 second period.

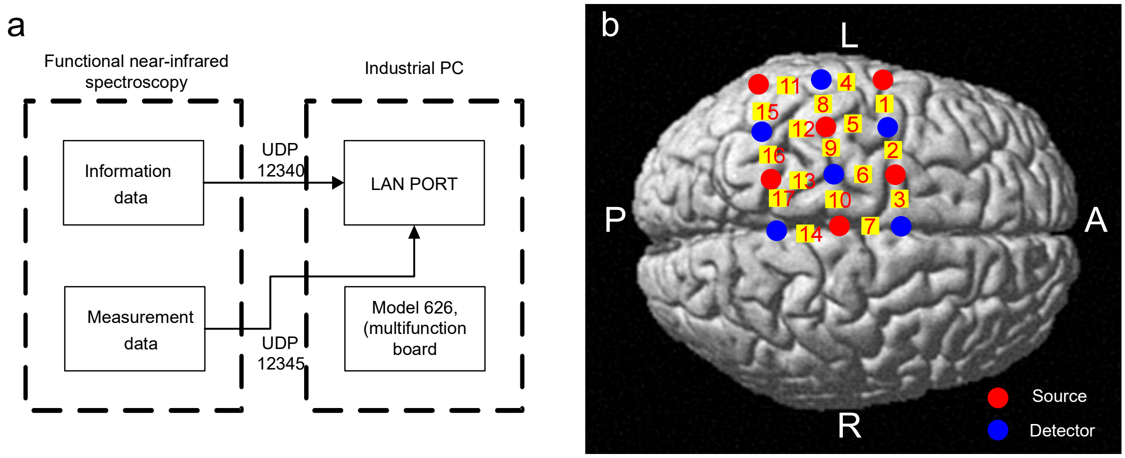

Figure S4. fNIRS and industrial PC connections, and channel placement: (a) fNIRS and industrial PC connections. fNIRS provides information data, which is about the state of fNIRS, and measurement data. These two types of data are transferred to industrial PC through UDP; (b) Channel placement. Using 6 sources and 6 detectors, 17 channels were generated. The abbreviation, A, P, L, and R mean anterior, posterior, left, and right respectively.

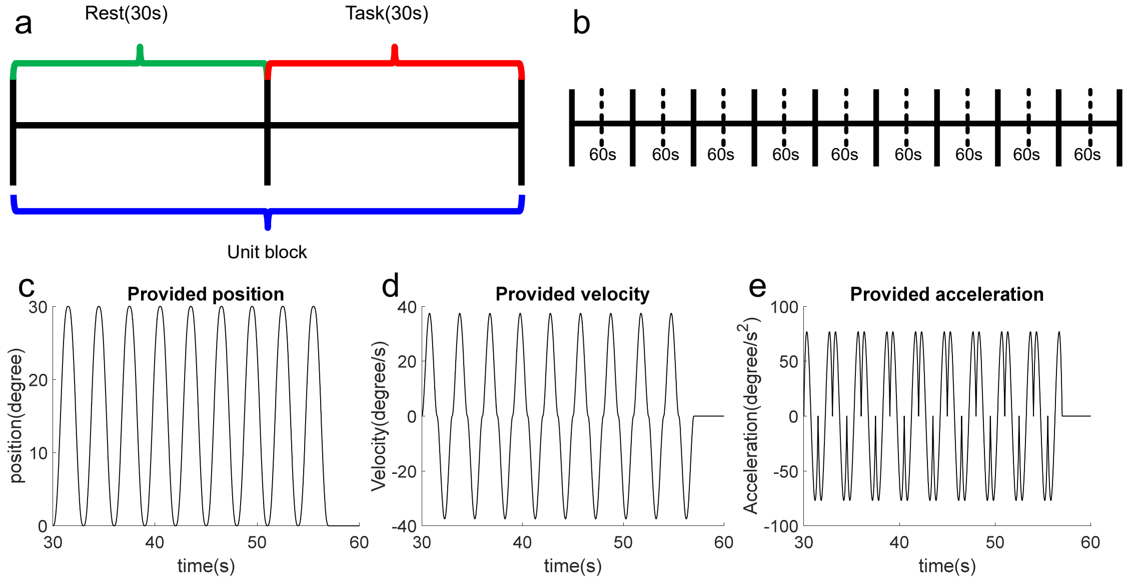

Figure S5. Block design and provided visual guide trajectory during the task period: (a) Unit block. One set consists of a 30 second rest period and a 30 second task period; (b) Total block design. It consists of 9 blocks to conduct 9 conditions; (c, d, e) Provided visual guide position, velocity, and acceleration trajectory at the task period.
