# Supplementary material for: Entropy Could Quantify Brain Activation Induced by Mechanical Impedance-Restrained Active Arm Motion: A Functional NIRS Study
Source: Entropy (Basel). 2022 Apr 15;24(4):556. doi: 10.3390/e24040556 (PMC9024511; doi:10.3390/e24040556)
Supplement: Supplementary file 1 [file entropy-24-00556-s001.zip › Table_S.pdf]

Table S1. Entropy ~ State+(1|subject) , 780 nm optical density data statistical results.

CH	Parameter	Fixed effect				Estimated intercept	Random effect			Error		
		Effect size	CI(95%) Lower	CI(95%) Upper	p-value		SD	CI(95%) Lower	CI(95%) Upper	SD	CI(95%) Lower	CI(95%) Upper
1	State	-0.0015	-0.0038	0.0007	0.1818	0.0411	0.0100	0.0073	0.0137	0.0114	0.0106	0.0122
2	State	0.0011	-0.0012	0.0033	0.3527	0.0415	0.0094	0.0068	0.0129	0.0112	0.0105	0.0121
3	State	-0.0003	-0.0023	0.0016	0.7244	0.0265	0.0106	0.0078	0.0144	0.0098	0.0091	0.0105
4	State	-0.0020	-0.0041	0.0002	0.0686	0.0341	0.0069	0.0049	0.0097	0.0108	0.0100	0.0116
5	State	0.0015	-0.0008	0.0038	0.2039	0.0431	0.0085	0.0061	0.0117	0.0116	0.0108	0.0125
6	State	0.0048	0.0024	0.0071	0.0001	0.0367	0.0096	0.0070	0.0133	0.0117	0.0109	0.0126
7	State	-0.0012	-0.0033	0.0009	0.2646	0.0357	0.0082	0.0059	0.0113	0.0107	0.0100	0.0115
8	State	0.0005	-0.0014	0.0024	0.5917	0.0283	0.0067	0.0048	0.0093	0.0097	0.0090	0.0104
9	State	0.0005	-0.0016	0.0025	0.6520	0.0330	0.0080	0.0058	0.0111	0.0102	0.0095	0.0110
10	State	0.0007	-0.0015	0.0029	0.5365	0.0372	0.0101	0.0073	0.0138	0.0111	0.0103	0.0119
11	State	-0.0001	-0.0019	0.0018	0.9564	0.0302	0.0059	0.0042	0.0082	0.0095	0.0088	0.0102
12	State	-0.0003	-0.0022	0.0016	0.7742	0.0296	0.0063	0.0045	0.0087	0.0096	0.0090	0.0104
13	State	0.0012	-0.0010	0.0034	0.2813	0.0361	0.0075	0.0054	0.0104	0.0110	0.0103	0.0119
14	State	0.0004	-0.0017	0.0026	0.6956	0.0405	0.0102	0.0075	0.0140	0.0110	0.0102	0.0118
15	State	-0.0004	-0.0024	0.0017	0.7258	0.0327	0.0073	0.0053	0.0102	0.0104	0.0097	0.0112
16	State	0.0001	-0.0019	0.0021	0.9317	0.0367	0.0091	0.0066	0.0125	0.0103	0.0095	0.0110
17	State	0.0001	-0.0022	0.0024	0.9314	0.0460	0.0111	0.0081	0.0152	0.0116	0.0108	0.0125

SD: standard deviation, CI: confident interval, the state consists of the rest state and task state, The value of the rest state is 0, and the value of the task state is 1, df = 18 (the number of period) *22 (the number of participant) -2 (the number of estimated variable) = 394, Significance is identified by CI not including 0 and p-value < 0.05, Red shade means that identified significance exists in all wavelength data.

Table S2. Entropy ~ State+(1|subject) , 805 nm optical density data statistical results.

CH	Parameter	Fixed effect				Estimated intercept	Random effect			Error		
		Effect size	CI(95%) Lower	CI(95%) Upper	p-value		SD	CI(95%) Lower	CI(95%) Upper	SD	CI(95%) Lower	CI(95%) Upper
1	State	-0.0014	-0.0036	0.0008	0.2217	0.0406	0.0084	0.0060	0.0116	0.0112	0.0104	0.0121
2	State	0.0018	-0.0005	0.0040	0.1234	0.0407	0.0085	0.0061	0.0117	0.0113	0.0105	0.0121
3	State	0.0004	-0.0015	0.0023	0.6678	0.0256	0.0096	0.0070	0.0131	0.0097	0.0090	0.0104
4	State	-0.0017	-0.0037	0.0002	0.0751	0.0322	0.0055	0.0039	0.0078	0.0098	0.0091	0.0105
5	State	-0.0002	-0.0025	0.0020	0.8462	0.0445	0.0087	0.0063	0.0120	0.0113	0.0105	0.0122
6	State	0.0029	0.0006	0.0052	0.0144	0.0357	0.0079	0.0057	0.0110	0.0116	0.0108	0.0124
7	State	-0.0017	-0.0036	0.0003	0.0900	0.0363	0.0078	0.0056	0.0107	0.0098	0.0091	0.0105
8	State	0.0000	-0.0018	0.0017	0.9791	0.0284	0.0052	0.0037	0.0074	0.0089	0.0083	0.0096
9	State	0.0013	-0.0008	0.0034	0.2222	0.0339	0.0073	0.0052	0.0101	0.0107	0.0100	0.0115
10	State	0.0030	0.0008	0.0052	0.0086	0.0376	0.0090	0.0065	0.0124	0.0113	0.0105	0.0121
11	State	-0.0002	-0.0021	0.0017	0.8412	0.0290	0.0049	0.0034	0.0070	0.0095	0.0088	0.0102
12	State	0.0009	-0.0011	0.0029	0.3889	0.0290	0.0061	0.0043	0.0086	0.0102	0.0095	0.0110
13	State	0.0005	-0.0015	0.0025	0.6177	0.0349	0.0063	0.0045	0.0089	0.0102	0.0095	0.0109
14	State	0.0000	-0.0021	0.0021	0.9937	0.0414	0.0096	0.0070	0.0131	0.0107	0.0100	0.0115
15	State	-0.0003	-0.0022	0.0017	0.7652	0.0308	0.0056	0.0039	0.0079	0.0099	0.0092	0.0106
16	State	0.0007	-0.0013	0.0028	0.4914	0.0361	0.0091	0.0066	0.0125	0.0103	0.0096	0.0111
17	State	0.0000	-0.0021	0.0022	0.9804	0.0443	0.0107	0.0078	0.0147	0.0110	0.0102	0.0118

SD: standard deviation, CI: confident interval, the state consists of the rest state and task state, The value of the rest state is 0, and the value of the task state is 1, df = 18 (the number of period) *22 (the number of participant) -2 (the number of estimated variable) = 394, Significance is identified by CI not including 0 and p-value < 0.05, Red shade means that identified significance exists in all wavelength data.

Table S3. Entropy ~ State+(1|subject) , 830 nm optical density data statistical results.

CH	Parameter	Fixed effect				Estimated intercept	Random effect			Error		
		Effect size	CI(95%) Lower	CI(95%) Upper	p-value		SD	CI(95%) Lower	CI(95%) Upper	SD	CI(95%) Lower	CI(95%) Upper
1	State	-0.0009	-0.0030	0.0012	0.3927	0.0351	0.0073	0.0053	0.0102	0.0105	0.0097	0.0112
2	State	0.0020	-0.0001	0.0040	0.0576	0.0341	0.0080	0.0058	0.0110	0.0103	0.0096	0.0110
3	State	-0.0001	-0.0018	0.0015	0.8942	0.0227	0.0075	0.0055	0.0103	0.0083	0.0077	0.0089
4	State	-0.0016	-0.0035	0.0002	0.0739	0.0290	0.0043	0.0030	0.0062	0.0092	0.0085	0.0098
5	State	0.0020	-0.0001	0.0042	0.0651	0.0389	0.0073	0.0052	0.0102	0.0110	0.0102	0.0118
6	State	0.0024	0.0004	0.0045	0.0213	0.0307	0.0065	0.0047	0.0091	0.0104	0.0097	0.0112
7	State	-0.0010	-0.0028	0.0008	0.2631	0.0299	0.0058	0.0041	0.0081	0.0090	0.0084	0.0096
8	State	-0.0002	-0.0018	0.0014	0.8484	0.0261	0.0045	0.0032	0.0064	0.0081	0.0075	0.0087
9	State	0.0000	-0.0017	0.0018	0.9913	0.0294	0.0053	0.0038	0.0075	0.0089	0.0083	0.0096
10	State	0.0002	-0.0018	0.0022	0.8658	0.0314	0.0076	0.0055	0.0105	0.0102	0.0095	0.0110
11	State	-0.0009	-0.0027	0.0008	0.3036	0.0276	0.0050	0.0036	0.0071	0.0090	0.0084	0.0097
12	State	0.0001	-0.0016	0.0018	0.9243	0.0267	0.0045	0.0032	0.0065	0.0086	0.0080	0.0092

13	State	0.0002	-0.0015	0.0020	0.7995	0.0303	0.0054	0.0038	0.0076	0.0088	0.0082	0.0095
14	State	-0.0012	-0.0032	0.0008	0.2547	0.0349	0.0076	0.0055	0.0105	0.0102	0.0095	0.0110
15	State	-0.0008	-0.0027	0.0010	0.3723	0.0278	0.0041	0.0028	0.0060	0.0093	0.0087	0.0100
16	State	-0.0002	-0.0020	0.0017	0.8622	0.0325	0.0077	0.0056	0.0106	0.0095	0.0088	0.0102
17	State	-0.0002	-0.0023	0.0018	0.8126	0.0402	0.0102	0.0074	0.0139	0.0104	0.0097	0.0112

SD: standard deviation, CI: confident interval, the state consists of the rest state and task state, The value of the rest state is 0, and the value of the task state is 1, df = 18 (the number of period) *22 (the number of participant) -2 (the number of estimated variable) = 394, Significance is identified by CI not including 0 and p-value < 0.05, Red shade means that identified significance exists in all wavelength density data.

Table S4. Entropy ~ Rest duration+ (1|subject) , 780 nm optical density data statistical results.

CH	Parameter	Fixed effect				Estimated intercept	Random effect			Error		
		Effect size*	CI(95%)		p-value		SD	CI(95%)		SD	CI(95%)	
			Lower*	Upper*				Lower	Upper		Lower	Upper
1	RD	0.0588	-0.2317	0.3492	0.6904	0.0405	0.0103	0.0074	0.0143	0.0107	0.0096	0.0119
2	RD	0.1366	-0.1465	0.4197	0.3426	0.0403	0.0108	0.0078	0.0149	0.0104	0.0094	0.0116
3	RD	-0.2010	-0.4611	0.0591	0.1291	0.0283	0.0115	0.0083	0.0158	0.0096	0.0086	0.0106
4	RD	0.0167	-0.2835	0.3168	0.9129	0.0339	0.0062	0.0041	0.0092	0.0111	0.0100	0.0123
5	RD	-0.0977	-0.4069	0.2115	0.5339	0.0440	0.0078	0.0054	0.0112	0.0114	0.0103	0.0126
6	RD	0.0143	-0.2703	0.2988	0.9213	0.0366	0.0094	0.0067	0.0132	0.0105	0.0094	0.0116
7	RD	0.0369	-0.2427	0.3165	0.7951	0.0354	0.0092	0.0066	0.0129	0.0103	0.0093	0.0114
8	RD	-0.0568	-0.3116	0.1980	0.6609	0.0288	0.0066	0.0046	0.0095	0.0094	0.0085	0.0104
9	RD	-0.0920	-0.3665	0.1825	0.5094	0.0338	0.0076	0.0054	0.0109	0.0101	0.0091	0.0112
10	RD	-0.0034	-0.2646	0.2578	0.9795	0.0372	0.0118	0.0086	0.0163	0.0096	0.0087	0.0107
11	RD	-0.1842	-0.4562	0.0877	0.1831	0.0319	0.0052	0.0034	0.0079	0.0100	0.0090	0.0111
12	RD	-0.0899	-0.3556	0.1758	0.5054	0.0304	0.0063	0.0043	0.0092	0.0098	0.0088	0.0109
13	RD	-0.0598	-0.3480	0.2284	0.6828	0.0366	0.0071	0.0049	0.0103	0.0106	0.0096	0.0118
14	RD	-0.1215	-0.4065	0.1636	0.4017	0.0416	0.0108	0.0078	0.0150	0.0105	0.0095	0.0117
15	RD	-0.2471	-0.5459	0.0517	0.1046	0.0349	0.0074	0.0051	0.0107	0.0110	0.0099	0.0122
16	RD	-0.1576	-0.4015	0.0863	0.2039	0.0381	0.0102	0.0074	0.0141	0.0090	0.0081	0.0100
17	RD	0.1103	-0.2001	0.4206	0.4844	0.0450	0.0117	0.0085	0.0163	0.0114	0.0103	0.0127

RD: rest duration, $\bullet^{*}: \times 10^{-3}$, SD: standard deviation, CI: confident interval, Entropy calculated from the rest state only, df = 9 (the number of period) *22 (the number of participant) -2 (the number of estimated variable) = 196, Significance is identified by CI not including 0 and p-value < 0.05

Table S5. Entropy ~ Rest duration+ (1|subject) , 805 nm optical density data statistical results.

CH	Parameter	Fixed effect				Estimated intercept	Random effect			Error		
		Effect size*	CI(95%)		p-value		SD	CI(95%)		SD	CI(95%)	
			Lower*	Upper*				Lower	Upper		Lower	Upper
1	RD	0.2614	-0.0347	0.5576	0.0833	0.0383	0.0090	0.0063	0.0126	0.0109	0.0098	0.0121
2	RD	-0.1260	-0.4414	0.1894	0.4318	0.0418	0.0087	0.0061	0.0124	0.0116	0.0105	0.0129
3	RD	-0.1233	-0.3739	0.1272	0.3329	0.0267	0.0102	0.0074	0.0141	0.0092	0.0083	0.0102
4	RD	0.2004	-0.0629	0.4636	0.1350	0.0304	0.0052	0.0035	0.0079	0.0097	0.0087	0.0108
5	RD	0.0296	-0.2939	0.3530	0.8571	0.0442	0.0082	0.0057	0.0118	0.0119	0.0107	0.0132
6	RD	-0.1395	-0.4426	0.1637	0.3654	0.0369	0.0087	0.0061	0.0123	0.0112	0.0101	0.0124
7	RD	0.1827	-0.0686	0.4341	0.1533	0.0347	0.0096	0.0069	0.0133	0.0093	0.0083	0.0103
8	RD	0.1093	-0.1333	0.3519	0.3754	0.0274	0.0052	0.0035	0.0077	0.0089	0.0081	0.0099
9	RD	0.2223	-0.0454	0.4901	0.1031	0.0319	0.0083	0.0059	0.0117	0.0099	0.0089	0.0110
10	RD	-0.0212	-0.2807	0.2383	0.8722	0.0378	0.0104	0.0075	0.0144	0.0096	0.0086	0.0106
11	RD	-0.0674	-0.3308	0.1960	0.6144	0.0296	0.0049	0.0032	0.0075	0.0097	0.0087	0.0108
12	RD	0.1229	-0.1652	0.4111	0.4011	0.0279	0.0055	0.0036	0.0084	0.0106	0.0096	0.0118
13	RD	0.0788	-0.1862	0.3438	0.5582	0.0342	0.0062	0.0042	0.0090	0.0098	0.0088	0.0108
14	RD	0.0389	-0.2357	0.3135	0.7801	0.0411	0.0104	0.0075	0.0144	0.0101	0.0091	0.0112
15	RD	-0.0122	-0.2811	0.2567	0.9289	0.0309	0.0054	0.0036	0.0081	0.0099	0.0089	0.0110
16	RD	-0.0246	-0.2965	0.2474	0.8587	0.0364	0.0092	0.0066	0.0129	0.0100	0.0090	0.0111
17	RD	-0.0088	-0.3005	0.2829	0.9524	0.0444	0.0116	0.0084	0.0160	0.0107	0.0097	0.0119

RD: rest duration, $\bullet^{*}: \times 10^{-3}$, SD: standard deviation, CI: confident interval, Entropy calculated from the rest state only, df = 9 (the number of period) *22 (the number of participant) -2 (the number of estimated variable) = 196, Significance is identified by CI not including 0 and p-value < 0.05

Table S6. Entropy ~ Rest duration+ (1|subject) , 830 nm optical density data statistical results.

CH	Parameter	Fixed effect				Estimated intercept	Random effect			Error		
		Effect size*	CI(95%)		p-value		SD	CI(95%)		SD	CI(95%)	
			Lower*	Upper*				Lower	Upper		Lower	Upper
1	RD	0.1549	-0.1239	0.4338	0.2746	0.0337	0.0078	0.0055	0.0111	0.0103	0.0093	0.0114
2	RD	-0.0148	-0.2666	0.2369	0.9077	0.0342	0.0084	0.0060	0.0117	0.0093	0.0084	0.0103
3	RD	-0.0957	-0.3100	0.1185	0.3791	0.0235	0.0087	0.0063	0.0121	0.0079	0.0071	0.0088
4	RD	0.2276	-0.0125	0.4677	0.0630	0.0270	0.0044	0.0029	0.0068	0.0088	0.0080	0.0098
5	RD	0.0695	-0.2322	0.3712	0.6502	0.0383	0.0067	0.0045	0.0098	0.0111	0.0100	0.0123
6	RD	-0.1509	-0.4286	0.1268	0.2853	0.0320	0.0067	0.0046	0.0097	0.0102	0.0092	0.0114
7	RD	0.2664	0.0360	0.4969	0.0237	0.0275	0.0064	0.0045	0.0091	0.0085	0.0076	0.0094
8	RD	0.1810	-0.0395	0.4013	0.1070	0.0245	0.0048	0.0033	0.0071	0.0081	0.0073	0.0090
9	RD	0.0639	-0.1715	0.2992	0.5931	0.0288	0.0057	0.0039	0.0083	0.0087	0.0078	0.0096
10	RD	0.0527	-0.1968	0.3021	0.6776	0.0309	0.0095	0.0069	0.0132	0.0092	0.0083	0.0102
11	RD	-0.0484	-0.3056	0.2088	0.7111	0.0281	0.0056	0.0037	0.0082	0.0095	0.0085	0.0105
12	RD	0.0023	-0.2294	0.2340	0.9844	0.0267	0.0045	0.0030	0.0068	0.0085	0.0077	0.0095
13	RD	-0.0001	-0.2256	0.2254	0.9993	0.0303	0.0059	0.0041	0.0085	0.0083	0.0075	0.0092
14	RD	0.0215	-0.2480	0.2910	0.8751	0.0348	0.0085	0.0060	0.0119	0.0099	0.0089	0.0110
15	RD	-0.0483	-0.3019	0.2054	0.7079	0.0282	0.0051	0.0034	0.0076	0.0093	0.0084	0.0104
16	RD	0.0358	-0.1960	0.2675	0.7611	0.0321	0.0081	0.0058	0.0112	0.0085	0.0077	0.0095
17	RD	0.1890	-0.0843	0.4623	0.1743	0.0385	0.0102	0.0074	0.0142	0.0101	0.0091	0.0112

RD: rest duration, $\bullet^{*}: \times 10^{-3}$, SD: standard deviation, CI: confident interval, Entropy calculated from the rest state only, df = 9 (the number of period) *22 (the number of participant) -2 (the number of estimated variable) = 196, Significance is identified by CI not including 0 and p-value < 0.05, Gray shade means that identified significance exists only in this 830 nm wavelength density data.

Table S7. Entropy ~ Task duration+ ω_n + ζ +(1|subject), 780 nm optical density data statistical results.

CH	Parameter	Fixed effect				Estimated intercept	Random effect			Error		
		Effect size*	CI(95%)		p-value		SD	CI(95%)		SD	CI(95%)	
			Lower*	Upper*				Lower	Upper		Lower	Upper
1	NF	-1.1429	-2.1869	-0.0990	0.0321	0.0378	0.0093	0.0066	0.0132	0.0120	0.0108	0.0133
	DR	0.8810	-3.2294	4.9913	0.6730							
	TD	0.4318	0.1012	0.7625	0.0107							
2	NF	0.4231	-0.5794	1.4257	0.4062	0.0407	0.0083	0.0058	0.0119	0.0115	0.0103	0.0127
	DR	-2.0181	-5.9655	1.9292	0.3146							
	TD	0.2635	-0.0541	0.5810	0.1034							
3	NF	-0.8985	-1.7501	-0.0469	0.0388	0.0290	0.0097	0.0070	0.0135	0.0098	0.0088	0.0108
	DR	-1.5803	-4.9334	1.7727	0.3538							
	TD	0.1448	-0.1250	0.4145	0.2912							
4	NF	-0.7752	-1.6575	0.1070	0.0847	0.0304	0.0078	0.0055	0.0111	0.0101	0.0091	0.0112
	DR	0.2879	-3.1859	3.7616	0.8703							
	TD	0.3764	0.0970	0.6558	0.0085							
5	NF	-1.0540	-2.0512	-0.0567	0.0384	0.0440	0.0091	0.0064	0.0129	0.0114	0.0103	0.0127
	DR	-1.7400	-5.6664	2.1865	0.3832							
	TD	0.5538	0.2380	0.8697	0.0007							
6	NF	-0.8593	-1.9512	0.2327	0.1223	0.0408	0.0099	0.0070	0.0141	0.0125	0.0113	0.0139
	DR	-1.1879	-5.4872	3.1115	0.5864							
	TD	0.4456	0.0997	0.7914	0.0118							
7	NF	-1.0392	-1.9925	-0.0859	0.0328	0.0346	0.0072	0.0050	0.0105	0.0109	0.0098	0.0121
	DR	0.4223	-3.3313	4.1758	0.8247							
	TD	0.2566	-0.0453	0.5585	0.0953							
8	NF	0.0699	-0.7215	0.8613	0.8619	0.0242	0.0076	0.0054	0.0108	0.0091	0.0082	0.0101
	DR	0.7646	-2.3516	3.8807	0.6290							
	TD	0.3666	0.1160	0.6173	0.0044							
9	NF	-0.5952	-1.4681	0.2778	0.1803	0.0338	0.0086	0.0061	0.0121	0.0100	0.0090	0.0111
	DR	-1.5406	-4.9777	1.8965	0.3778							
	TD	0.2968	0.0203	0.5733	0.0355							
10	NF	-0.6627	-1.6996	0.3742	0.2090	0.0406	0.0086	0.0060	0.0123	0.0119	0.0107	0.0132
	DR	-0.6899	-4.7725	3.3927	0.7393							
	TD	-0.0100	-0.3384	0.3184	0.9523							
11	NF	0.4687	-0.2827	1.2202	0.2201	0.0298	0.0068	0.0048	0.0096	0.0086	0.0078	0.0096
	DR	-1.2626	-4.2213	1.6961	0.4010							
	TD	0.0229	-0.2151	0.2609	0.8494							
12	NF	-0.4080	-1.2065	0.3905	0.3148	0.0296	0.0064	0.0045	0.0093	0.0091	0.0082	0.0101
	DR	-1.9222	-5.0661	1.2216	0.2293							
	TD	0.2875	0.0346	0.5404	0.0261							

13	NF	-0.9539	-1.8752	-0.0325	0.0425	0.0367	0.0082	0.0058	0.0117	0.0105	0.0095	0.0117
	DR	-2.8674	-6.4951	0.7602	0.1206							
	TD	0.6352	0.3434	0.9269	0.0000							
14	NF	-0.5799	-1.5522	0.3924	0.2409	0.0419	0.0098	0.0070	0.0138	0.0111	0.0100	0.0124
	DR	-1.0973	-4.9255	2.7309	0.5725							
	TD	0.1882	-0.1197	0.4962	0.2294							
15	NF	0.0287	-0.8204	0.8778	0.9470	0.0343	0.0071	0.0050	0.0102	0.0097	0.0088	0.0108
	DR	-2.8389	-6.1821	0.5044	0.0956							
	TD	0.0789	-0.1901	0.3478	0.5637							
16	NF	-1.0818	-2.0282	-0.1354	0.0253	0.0384	0.0083	0.0058	0.0118	0.0108	0.0098	0.0120
	DR	-0.7526	-4.4790	2.9738	0.6908							
	TD	0.2383	-0.0614	0.5381	0.1185							
17	NF	-0.9423	-1.8979	0.0133	0.0532	0.0490	0.0110	0.0080	0.0153	0.0109	0.0099	0.0121
	DR	-3.4875	-7.2501	0.2750	0.0691							
	TD	0.3387	0.0360	0.6413	0.0285							

NF: natural frequency, DR: damping ratio, TD: task duration, $\bullet^*$: $\times 10^{-3}$, SD: standard deviation, CI: confident interval, df = 9 (the number of period) *22 (the number of participant) -4 (the number of estimated variable) = 194, Significance is identified by CI not including 0 and p-value < 0.05, Gray shade means that identified significance exists only in this 780 nm optical density data, Blue shade means that identified significance exists not only in this 780 nm wavelength data but also in 805 or 830nm wavelength data, Red shade means that identified significance exists in all wavelength data.

Table S8. Entropy ~ Task duration+ω_n + ζ+(1|subject), 805 nm optical density data statistical results.

CH	Parameter	Fixed effect				Estimated intercept	Random effect			Error		
		Effect size*	CI(95%)		p-value		SD	CI(95%)		SD	CI(95%)	
			Lower*	Upper*				Lower	Upper		Lower	Upper
1	NF	-1.1459	-2.1427	-0.1491	0.0245	0.0442	0.0075	0.0052	0.0109	0.0114	0.0103	0.0127
	DR	-2.2554	-6.1802	1.6695	0.2585							
	TD	0.0713	-0.2445	0.3870	0.6567							
2	NF	-0.3528	-1.3100	0.6044	0.4682	0.0421	0.0081	0.0057	0.0116	0.0110	0.0099	0.0122
	DR	-0.4017	-4.1706	3.3672	0.8337							
	TD	0.1818	-0.1213	0.4850	0.2383							
3	NF	-0.7273	-1.6071	0.1525	0.1047	0.0291	0.0088	0.0063	0.0124	0.0101	0.0091	0.0112
	DR	-0.4613	-3.9254	3.0027	0.7931							
	TD	-0.0409	-0.3195	0.2378	0.7727							
4	NF	-0.7141	-1.5309	0.1027	0.0862	0.0324	0.0058	0.0039	0.0085	0.0094	0.0084	0.0104
	DR	-3.6693	-6.8851	-0.4534	0.0256							
	TD	0.3840	0.1253	0.6426	0.0038							
5	NF	-0.2829	-1.2218	0.6560	0.5530	0.0431	0.0089	0.0063	0.0125	0.0108	0.0097	0.0119
	DR	-0.8547	-4.5516	2.8422	0.6489							
	TD	0.2854	-0.0120	0.5828	0.0599							
6	NF	-1.4137	-2.4112	-0.4162	0.0057	0.0420	0.0075	0.0052	0.0109	0.0114	0.0103	0.0127
	DR	-1.0171	-4.9445	2.9104	0.6101							
	TD	0.1807	-0.1352	0.4967	0.2606							
7	NF	-0.6976	-1.5375	0.1424	0.1031	0.0369	0.0062	0.0043	0.0090	0.0096	0.0087	0.0107
	DR	-0.4823	-3.7896	2.8249	0.7739							
	TD	0.0396	-0.2264	0.3056	0.7694							
8	NF	-0.0478	-0.7903	0.6947	0.8991	0.0247	0.0055	0.0038	0.0080	0.0085	0.0077	0.0094
	DR	-0.2224	-3.1458	2.7010	0.8809							
	TD	0.3976	0.1625	0.6328	0.0010							
9	NF	-0.8545	-1.8158	0.1068	0.0812	0.0344	0.0065	0.0044	0.0096	0.0110	0.0099	0.0122
	DR	0.4189	-3.3661	4.2039	0.8275							
	TD	0.3024	-0.0021	0.6068	0.0516							
10	NF	-1.3942	-2.4600	-0.3284	0.0106	0.0435	0.0080	0.0055	0.0116	0.0122	0.0110	0.0135
	DR	-0.1031	-4.2996	4.0933	0.9614							
	TD	0.1334	-0.2042	0.4709	0.4368							
11	NF	0.7252	-0.0412	1.4915	0.0635	0.0267	0.0054	0.0037	0.0080	0.0088	0.0079	0.0097
	DR	-1.3963	-4.4137	1.6211	0.3626							
	TD	0.1280	-0.1148	0.3707	0.2998							
12	NF	-0.3368	-1.1910	0.5174	0.4377	0.0310	0.0065	0.0045	0.0094	0.0098	0.0088	0.0109
	DR	-2.1673	-5.5306	1.1960	0.2053							
	TD	0.2039	-0.0667	0.4744	0.1389							
13	NF	-0.5726	-1.5007	0.3554	0.2251	0.0370	0.0063	0.0043	0.0093	0.0106	0.0096	0.0118
	DR	-0.9849	-4.6390	2.6691	0.5956							
	TD	0.1161	-0.1778	0.4101	0.4367							
14	NF	-1.5632	-2.4999	-0.6265	0.0012	0.0449	0.0090	0.0064	0.0127	0.0107	0.0097	0.0119
	DR	-0.4388	-4.1269	3.2493	0.8147							
	TD	0.1677	-0.1290	0.4643	0.2664							
15	NF	-0.3621	-1.2257	0.5015	0.4093	0.0344	0.0055	0.0037	0.0083	0.0099	0.0089	0.0110
	DR	-3.1823	-6.5827	0.2180	0.0664							
	TD	0.0341	-0.2394	0.3076	0.8059							
16	NF	-0.8043	-1.7051	0.0965	0.0798	0.0416	0.0091	0.0065	0.0128	0.0103	0.0093	0.0115

CH	Parameter	Effect size*	Lower*	Upper*	p-value	Estimated intercept	SD	Lower	Upper	SD	Lower	Upper
	DR	-3.6059	-7.1527	-0.0591	0.0463							
	TD	0.1268	-0.1585	0.4121	0.3818							
17	NF	-0.9102	-1.8622	0.0417	0.0608	0.0465	0.0099	0.0071	0.0139	0.0109	0.0098	0.0121
	DR	0.2291	-3.5191	3.9773	0.9042							
	TD	0.0402	-0.2613	0.3417	0.7929							

NF: natural frequency, DR: damping ratio, TD: task duration, $\bullet^{*}$: $\times 10^{-3}$, SD: standard deviation, CI: confident interval, df = 9 (the number of period) *22 (the number of participant) -4 (the number of estimated variable) = 194, Significance is identified by CI not including 0 and p-value < 0.05, Gray shade means that identified significance exists only in this 805 nm wavelength data, Blue shade means that identified significance exists not only in this 805 nm wavelength data but also in 780 or 830nm wavelength data, Red shade means that identified significance exists in all wavelength data.

Table S9. Entropy ~ Task duration+ω_n + ζ+(1|subject), 830 nm optical density data statistical results.

CH	Parameter	Fixed effect				Estimated intercept	Random effect			Error		
		Effect size*	CI(95%) Lower*	CI(95%) Upper*	p-value		SD	CI(95%) Lower	CI(95%) Upper	SD	CI(95%) Lower	CI(95%) Upper
1	NF	-0.5180	-1.4513	0.4154	0.2751	0.0373	0.0067	0.0045	0.0097	0.0107	0.0096	0.0119
	DR	-2.2619	-5.9368	1.4131	0.2263							
	TD	0.0695	-0.2261	0.3651	0.6433							
2	NF	-0.0179	-0.9773	0.9416	0.9707	0.0343	0.0078	0.0054	0.0112	0.0110	0.0099	0.0122
	DR	-0.4736	-4.2512	3.3041	0.8050							
	TD	0.2261	-0.0778	0.5299	0.1439							
3	NF	-0.5136	-1.2677	0.2405	0.1808	0.0232	0.0061	0.0043	0.0088	0.0086	0.0078	0.0096
	DR	0.7877	-2.1815	3.7568	0.6014							
	TD	0.0123	-0.2265	0.2511	0.9193							
4	NF	-0.8239	-1.5891	-0.0587	0.0350	0.0297	0.0046	0.0031	0.0070	0.0088	0.0079	0.0097
	DR	-3.4965	-6.5094	-0.4836	0.0232							
	TD	0.3606	0.1183	0.6030	0.0037							
5	NF	-0.6322	-1.5670	0.3027	0.1839	0.0398	0.0078	0.0055	0.0112	0.0107	0.0096	0.0119
	DR	-0.3037	-3.9846	3.3772	0.8709							
	TD	0.3343	0.0382	0.6304	0.0271							
6	NF	-0.9333	-1.8432	-0.0233	0.0445	0.0334	0.0062	0.0042	0.0092	0.0104	0.0094	0.0116
	DR	-1.4144	-4.9972	2.1684	0.4372							
	TD	0.3963	0.1082	0.6845	0.0073							
7	NF	-0.7009	-1.5204	0.1185	0.0932	0.0308	0.0048	0.0032	0.0074	0.0094	0.0085	0.0104
	DR	0.0771	-3.1495	3.3036	0.9625							
	TD	0.0092	-0.2503	0.2688	0.9442							
8	NF	-0.0138	-0.6620	0.6344	0.9666	0.0233	0.0047	0.0032	0.0069	0.0074	0.0067	0.0082
	DR	-0.9289	-3.4810	1.6233	0.4737							
	TD	0.3621	0.1568	0.5673	0.0006							
9	NF	-0.7048	-1.4818	0.0722	0.0752	0.0282	0.0050	0.0033	0.0074	0.0089	0.0080	0.0099
	DR	0.1350	-2.9244	3.1943	0.9307							
	TD	0.3163	0.0702	0.5624	0.0120							
10	NF	-0.6814	-1.6207	0.2578	0.1541	0.0318	0.0057	0.0038	0.0086	0.0108	0.0097	0.0119
	DR	0.3880	-3.3100	4.0860	0.8363							
	TD	0.1424	-0.1551	0.4398	0.3464							
11	NF	0.5076	-0.1812	1.1964	0.1477	0.0239	0.0052	0.0036	0.0076	0.0079	0.0071	0.0088
	DR	-0.6079	-3.3200	2.1041	0.6589							
	TD	0.1861	-0.0321	0.4042	0.0941							
12	NF	-0.2131	-0.9622	0.5360	0.5754	0.0258	0.0044	0.0029	0.0068	0.0086	0.0077	0.0095
	DR	-0.9669	-3.9165	1.9826	0.5187							
	TD	0.2618	0.0245	0.4990	0.0308							
13	NF	-0.1818	-0.9722	0.6086	0.6506	0.0298	0.0051	0.0034	0.0076	0.0091	0.0082	0.0100
	DR	-0.5870	-3.6992	2.5252	0.7103							
	TD	0.1863	-0.0641	0.4366	0.1439							
14	NF	-0.4716	-1.3868	0.4436	0.3108	0.0339	0.0065	0.0045	0.0096	0.0105	0.0094	0.0116
	DR	-1.1622	-4.7657	2.4413	0.5255							
	TD	0.2426	-0.0473	0.5325	0.1004							
15	NF	-0.5074	-1.3467	0.3319	0.2346	0.0291				0.0096	0.0087	0.0106
	DR	-2.4197	-5.7243	0.8849	0.1503							
	TD	0.1755	-0.0903	0.4413	0.1943							
16	NF	-1.0418	-1.9317	-0.1519	0.0220	0.0362	0.0073	0.0051	0.0105	0.0102	0.0092	0.0113
	DR	-2.0809	-5.5848	1.4229	0.2429							
	TD	0.1280	-0.1539	0.4098	0.3717							
17	NF	-0.8872	-1.8136	0.0393	0.0604	0.0436	0.0101	0.0072	0.0140	0.0106	0.0096	0.0118
	DR	-2.8585	-6.5062	0.7892	0.1238							
	TD	0.1812	-0.1123	0.4746	0.2248							

NF: natural frequency, DR: damping ratio, TD: task duration, $\bullet^{*}$: $\times 10^{-3}$, SD: standard deviation, CI: confident interval, df = 9 (the number of period) *22 (the number of participant) -4 (the number of estimated variable) = 194, Significance is identified by CI not including 0 and p-value < 0.05, Gray shade means that identified significance exists only in this 830 nm wavelength data. Blue shade means that identified significance exists not only in this 830 nm wavelength data but also in 780 or 805nm wavelength data, Red shade means that identified significance exists in all wavelength data. Blank in random effect column means there is no random effect.

Table S10. Signal amplitude ~ State+(1|subject) , 780 nm optical density data statistical results.

CH	Parameter	Fixed effect				Estimated intercept	Random effect			Error		
		Effect size	CI(95%) Lower	CI(95%) Upper	p-value		SD	CI(95%) Lower	CI(95%) Upper	SD	CI(95%) Lower	CI(95%) Upper
1	State	0.0025	0.0013	0.0038	0.0001	-0.0013				0.0065	0.0061	0.0070
2	State	0.0014	0.0004	0.0023	0.0069	-0.0007				0.0050	0.0046	0.0053
3	State	0.0003	-0.0015	0.0021	0.7370	-0.0002				0.0093	0.0086	0.0099
4	State	0.0025	0.0013	0.0037	0.0000	-0.0013				0.0061	0.0057	0.0065
5	State	0.0010	-0.0002	0.0022	0.0911	-0.0005				0.0060	0.0056	0.0065
6	State	0.0000	-0.0016	0.0016	0.9918	0.0000				0.0081	0.0075	0.0086
7	State	0.0009	-0.0001	0.0019	0.0691	-0.0004				0.0049	0.0046	0.0053
8	State	0.0000	-0.0011	0.0012	0.9601	0.0000				0.0058	0.0054	0.0062
9	State	0.0001	-0.0010	0.0013	0.8048	-0.0001				0.0057	0.0053	0.0061
10	State	0.0016	0.0002	0.0029	0.0217	-0.0008				0.0067	0.0063	0.0072
11	State	0.0015	0.0005	0.0025	0.0039	-0.0007				0.0051	0.0047	0.0054
12	State	-0.0005	-0.0016	0.0006	0.3553	0.0003				0.0056	0.0052	0.0060
13	State	0.0010	0.0000	0.0020	0.0404	-0.0005				0.0051	0.0047	0.0054
14	State	0.0008	-0.0001	0.0018	0.0807	-0.0004				0.0048	0.0045	0.0052
15	State	0.0004	-0.0006	0.0015	0.4350	-0.0002				0.0053	0.0049	0.0056
16	State	0.0004	-0.0006	0.0014	0.4003	-0.0002				0.0050	0.0047	0.0054
17	State	0.0008	-0.0002	0.0018	0.1264	-0.0004				0.0050	0.0046	0.0053

SD: standard deviation, CI: confident interval, the state consists of the rest state and task state, The value of the rest state is 0, and the value of the task state is 1, df = 18 (the number of period) *22 (the number of participant) -2 (the number of estimated variable) = 394, Significance is identified by CI not including 0 and p-value < 0.05, Red shade means that identified significance exists in all wavelength data. There is no random effect.

Table S11. Signal amplitude ~ State+(1|subject) , 805 nm optical density data statistical results.

CH	Parameter	Fixed effect				Estimated intercept	Random effect			Error		
		Effect size	CI(95%) Lower	CI(95%) Upper	p-value		SD	CI(95%) Lower	CI(95%) Upper	SD	CI(95%) Lower	CI(95%) Upper
1	State	0.0038	0.0024	0.0052	0.0000	-0.0019				0.0069	0.0065	0.0074
2	State	0.0030	0.0020	0.0041	0.0000	-0.0015				0.0053	0.0050	0.0057
3	State	0.0022	0.0003	0.0040	0.0208	-0.0011				0.0093	0.0087	0.0100
4	State	0.0040	0.0027	0.0054	0.0000	-0.0020				0.0068	0.0064	0.0073
5	State	0.0028	0.0015	0.0041	0.0000	-0.0013				0.0066	0.0062	0.0071
6	State	0.0027	0.0012	0.0043	0.0005	-0.0014				0.0077	0.0072	0.0083
7	State	0.0013	0.0003	0.0023	0.0137	-0.0006				0.0052	0.0049	0.0056
8	State	0.0011	-0.0002	0.0023	0.0902	-0.0005				0.0063	0.0058	0.0067
9	State	0.0019	0.0007	0.0031	0.0023	-0.0009				0.0061	0.0057	0.0065
10	State	0.0020	0.0006	0.0034	0.0055	-0.0010				0.0070	0.0065	0.0075
11	State	0.0024	0.0013	0.0035	0.0000	-0.0012				0.0054	0.0051	0.0058
12	State	0.0005	-0.0008	0.0017	0.4597	-0.0002				0.0062	0.0058	0.0067
13	State	0.0025	0.0014	0.0036	0.0000	-0.0012				0.0057	0.0053	0.0061
14	State	0.0011	0.0001	0.0021	0.0397	-0.0005				0.0052	0.0048	0.0055
15	State	0.0012	0.0000	0.0023	0.0445	-0.0006				0.0058	0.0054	0.0062
16	State	0.0015	0.0003	0.0026	0.0108	-0.0007				0.0057	0.0054	0.0062
17	State	0.0020	0.0009	0.0032	0.0004	-0.0010				0.0057	0.0053	0.0061

SD: standard deviation, CI: confident interval, the state consists of the rest state and task state, The value of the rest state is 0, and the value of the task state is 1, df = 18 (the number of period) *22 (the number of participant) -2 (the number of estimated variable) = 394, Significance is identified by CI not including 0 and p-value < 0.05, Blue shade means that identified significance exists not only in this 805 nm wavelength data but also in 830nm wavelength data, Red shade means that identified significance exists in all wavelength data. There is no random effect.

Table S12. Signal amplitude ~ State+(1|subject) , 830 nm optical density data statistical results.

CH	Parameter	Fixed effect				Estimated intercept	Random effect			Error		
		Effect size	CI(95%) Lower	CI(95%) Upper	p-value		SD	CI(95%) Lower	CI(95%) Upper	SD	CI(95%) Lower	CI(95%) Upper
1	State	0.0045	0.0030	0.0060	0.0000	-0.0022				0.0077	0.0071	0.0082
2	State	0.0036	0.0025	0.0048	0.0000	-0.0018				0.0058	0.0054	0.0062
3	State	0.0028	0.0009	0.0046	0.0038	-0.0014				0.0094	0.0088	0.0101
4	State	0.0045	0.0030	0.0060	0.0000	-0.0022				0.0077	0.0072	0.0082
5	State	0.0035	0.0021	0.0049	0.0000	-0.0017				0.0070	0.0066	0.0075
6	State	0.0032	0.0016	0.0048	0.0001	-0.0016				0.0080	0.0074	0.0086

7	State	0.0017	0.0005	0.0028	0.0043	-0.0008				0.0057	0.0053	0.0061
8	State	0.0014	0.0001	0.0027	0.0361	-0.0007				0.0066	0.0062	0.0071
9	State	0.0024	0.0011	0.0037	0.0004	-0.0012				0.0066	0.0062	0.0071
10	State	0.0024	0.0009	0.0039	0.0013	-0.0012				0.0074	0.0069	0.0079
11	State	0.0027	0.0016	0.0039	0.0000	-0.0013				0.0059	0.0055	0.0063
12	State	0.0007	-0.0006	0.0021	0.2655	-0.0004				0.0066	0.0062	0.0071
13	State	0.0028	0.0016	0.0040	0.0000	-0.0014				0.0062	0.0058	0.0066
14	State	0.0013	0.0002	0.0024	0.0226	-0.0006				0.0056	0.0053	0.0060
15	State	0.0014	0.0002	0.0026	0.0243	-0.0007				0.0062	0.0058	0.0067
16	State	0.0018	0.0006	0.0030	0.0033	-0.0009				0.0061	0.0057	0.0065
17	State	0.0022	0.0010	0.0034	0.0003	-0.0011				0.0061	0.0057	0.0066

SD: standard deviation, CI: confident interval, the state consists of the rest state and task state, The value of the rest state is 0, and the value of the task state is 1, df = 18 (the number of period) *22 (the number of participant) -2 (the number of estimated variable) = 394, Significance is identified by CI not including 0 and p-value < 0.05, Gray shade means that identified significance exists only in this 830 nm wavelength data, Blue shade means that identified significance exists not only in this 830 nm wavelength data but also in 805nm wavelength data, Red shade means that identified significance exists in all wavelength density data. There is no random effect.

Table S13. Signal amplitude~ Rest duration+ (1|subject) , 780 nm optical density data statistical results.

CH	Parameter	Fixed effect				Estimated intercept	Random effect			Error		
		Effect size*	CI(95%)		p-value		SD	CI(95%)		SD	CI(95%)	
			Lower*	Upper*				Lower	Upper		Lower	Upper
1	RD	-0.1302	-0.3139	0.0535	0.1637	-0.0001				0.0068	0.0061	0.0075
2	RD	-0.0782	-0.2200	0.0636	0.2780	0.0000				0.0052	0.0047	0.0058
3	RD	-0.0899	-0.3750	0.1952	0.5347	0.0006				0.0105	0.0095	0.0116
4	RD	-0.0720	-0.2470	0.1029	0.4178	-0.0006				0.0064	0.0058	0.0071
5	RD	-0.1025	-0.2678	0.0629	0.2232	0.0004				0.0061	0.0055	0.0067
6	RD	-0.1488	-0.3966	0.0991	0.2379	0.0013				0.0091	0.0083	0.0101
7	RD	-0.0574	-0.1982	0.0833	0.4220	0.0001				0.0052	0.0047	0.0057
8	RD	-0.1272	-0.2841	0.0297	0.1114	0.0011				0.0058	0.0052	0.0064
9	RD	-0.1455	-0.3064	0.0154	0.0761	0.0012				0.0059	0.0054	0.0065
10	RD	-0.1239	-0.3315	0.0837	0.2405	0.0003				0.0076	0.0069	0.0084
11	RD	-0.0850	-0.2239	0.0539	0.2288	0.0000				0.0051	0.0046	0.0056
12	RD	-0.1054	-0.2577	0.0469	0.1740	0.0012				0.0056	0.0051	0.0062
13	RD	-0.1114	-0.2550	0.0322	0.1275	0.0005				0.0053	0.0048	0.0058
14	RD	-0.0544	-0.1927	0.0839	0.4388	0.0001				0.0051	0.0046	0.0056
15	RD	-0.0696	-0.2164	0.0772	0.3511	0.0004				0.0054	0.0049	0.0060
16	RD	-0.0391	-0.1806	0.1024	0.5865	0.0002				0.0052	0.0047	0.0058
17	RD	-0.0399	-0.1800	0.1003	0.5755	0.0000				0.0052	0.0047	0.0057

RD: rest duration, $\bullet^{*}$: $\times 10^{-3}$, SD: standard deviation, CI: confident interval, Signal amplitude calculated from the rest state only, df = 9 (the number of period) *22 (the number of participant) -2 (the number of estimated variable) = 196, Significance is identified by CI not including 0 and p-value < 0.05, There is no random effect.

Table S14. Signal amplitude~ Rest duration+ (1|subject) , 805 nm optical density data statistical results.

CH	Parameter	Fixed effect				Estimated intercept	Random effect			Error		
		Effect size*	CI(95%)		p-value		SD	CI(95%)		SD	CI(95%)	
			Lower*	Upper*				Lower	Upper		Lower	Upper
1	RD	-0.0968	-0.2922	0.0985	0.3293	-0.0010				0.0072	0.0065	0.0079
2	RD	-0.0666	-0.2105	0.0774	0.3631	-0.0009				0.0053	0.0048	0.0059
3	RD	-0.0950	-0.3814	0.1915	0.5140	-0.0002				0.0106	0.0096	0.0116
4	RD	-0.0493	-0.2423	0.1438	0.6151	-0.0015				0.0071	0.0064	0.0078
5	RD	-0.0906	-0.2700	0.0888	0.3204	-0.0005				0.0066	0.0060	0.0073
6	RD	-0.1489	-0.3807	0.0829	0.2068	0.0000				0.0085	0.0077	0.0094
7	RD	-0.0593	-0.2089	0.0904	0.4357	-0.0001				0.0055	0.0050	0.0061
8	RD	-0.1124	-0.2826	0.0578	0.1944	0.0005				0.0063	0.0057	0.0069
9	RD	-0.1208	-0.2923	0.0506	0.1662	0.0002				0.0063	0.0057	0.0070
10	RD	-0.1052	-0.3202	0.1098	0.3357	0.0000				0.0079	0.0072	0.0087
11	RD	-0.0799	-0.2306	0.0709	0.2973	-0.0005				0.0056	0.0050	0.0061
12	RD	-0.0944	-0.2659	0.0771	0.2790	0.0006				0.0063	0.0057	0.0070
13	RD	-0.0935	-0.2513	0.0643	0.2442	-0.0004				0.0058	0.0053	0.0064
14	RD	-0.0548	-0.2018	0.0922	0.4632	0.0000				0.0054	0.0049	0.0060
15	RD	-0.0444	-0.2096	0.1207	0.5964	-0.0002				0.0061	0.0055	0.0067
16	RD	-0.0218	-0.1810	0.1373	0.7868	-0.0005				0.0059	0.0053	0.0065
17	RD	-0.0522	-0.2077	0.1034	0.5090	-0.0005				0.0057	0.0052	0.0063

RD: rest duration, $\bullet^{*}$: $\times 10^{-3}$, SD: standard deviation, CI: confident interval, Signal amplitude calculated from the rest state only, df = 9 (the number of period) *22 (the number of participant) -2 (the number of estimated variable) = 196, Significance is identified by CI not including 0 and p-value < 0.05, There is no random effect.

Table S15. Signal amplitude ~ Rest duration+ (1|subject) , 830 nm optical density data statistical results.

CH	Parameter	Fixed effect				Estimated intercept	Random effect			Error		
		Effect size*	CI(95%) Lower*	CI(95%) Upper*	p-value		SD	CI(95%) Lower	CI(95%) Upper	SD	CI(95%) Lower	CI(95%) Upper
1	RD	-0.0998	-0.3130	0.1134	0.3572	-0.0013				0.0079	0.0071	0.0087
2	RD	-0.0771	-0.2332	0.0790	0.3313	-0.0011				0.0058	0.0052	0.0063
3	RD	-0.1047	-0.3930	0.1836	0.4747	-0.0004				0.0106	0.0096	0.0117
4	RD	-0.0493	-0.2649	0.1664	0.6528	-0.0018				0.0079	0.0072	0.0088
5	RD	-0.0913	-0.2800	0.0974	0.3413	-0.0009				0.0070	0.0063	0.0077
6	RD	-0.1467	-0.3845	0.0911	0.2252	-0.0003				0.0088	0.0079	0.0097
7	RD	-0.0555	-0.2185	0.1075	0.5026	-0.0003				0.0060	0.0054	0.0066
8	RD	-0.1042	-0.2830	0.0746	0.2519	0.0003				0.0066	0.0060	0.0073
9	RD	-0.1187	-0.3054	0.0680	0.2114	-0.0001				0.0069	0.0062	0.0076
10	RD	-0.1043	-0.3253	0.1167	0.3532	-0.0002				0.0081	0.0074	0.0090
11	RD	-0.0780	-0.2417	0.0858	0.3489	-0.0006				0.0060	0.0055	0.0067
12	RD	-0.0824	-0.2654	0.1005	0.3753	0.0004				0.0067	0.0061	0.0074
13	RD	-0.0826	-0.2517	0.0865	0.3365	-0.0006				0.0062	0.0056	0.0069
14	RD	-0.0420	-0.2005	0.1165	0.6022	-0.0003				0.0058	0.0053	0.0064
15	RD	-0.0448	-0.2218	0.1323	0.6186	-0.0003				0.0065	0.0059	0.0072
16	RD	-0.0183	-0.1858	0.1493	0.8301	-0.0007				0.0062	0.0056	0.0068
17	RD	-0.0529	-0.2188	0.1130	0.5303	-0.0006				0.0061	0.0055	0.0067

RD: rest duration, $\bullet^{*}$: $\times 10^{-3}$, SD: standard deviation, CI: confident interval, Signal amplitude calculated from the rest state only, df = 9 (the number of period) *22 (the number of participant) -2 (the number of estimated variable) = 196, Significance is identified by CI not including 0 and p-value < 0.05, There is no random effect

Table S16. Signal amplitude ~ Task duration+ω_n + ζ+(1|subject) , 780 nm optical density data statistical results.

CH	Parameter	Fixed effect				Estimated intercept	Random effect			Error		
		Effect size	CI(95%) Lower	CI(95%) Upper	p-value		SD	CI(95%) Lower	CI(95%) Upper	SD	CI(95%) Lower	CI(95%) Upper
1	NF	0.0006	0.0000	0.0011	0.0385	-0.0008				0.0061	0.0055	0.0067
	DR	0.0000	-0.0021	0.0021	0.9937							
	TD	0.0000	-0.0001	0.0002	0.6178							
2	NF	0.0004	0.0000	0.0008	0.0483	-0.0004				0.0046	0.0042	0.0051
	DR	-0.0004	-0.0020	0.0012	0.6197							
	TD	0.0000	-0.0001	0.0002	0.7174							
3	NF	0.0004	-0.0001	0.0010	0.1433	-0.0004	0.0041	0.0028	0.0060	0.0066	0.0060	0.0074
	DR	-0.0010	-0.0033	0.0013	0.3840							
	TD	0.0000	-0.0002	0.0002	0.8093							
4	NF	0.0007	0.0002	0.0012	0.0055	-0.0001				0.0056	0.0051	0.0062
	DR	-0.0005	-0.0024	0.0014	0.6045							
	TD	0.0000	-0.0002	0.0001	0.7815							
5	NF	0.0008	0.0003	0.0013	0.0025	-0.0024				0.0058	0.0052	0.0064
	DR	0.0001	-0.0018	0.0021	0.8886							
	TD	0.0000	-0.0001	0.0002	0.6266							
6	NF	0.0004	-0.0001	0.0009	0.1304	-0.0020	0.0037	0.0025	0.0053	0.0056	0.0050	0.0062
	DR	-0.0003	-0.0022	0.0017	0.7854							
	TD	0.0001	0.0000	0.0003	0.1646							
7	NF	0.0007	0.0003	0.0010	0.0002	-0.0008	0.0020	0.0013	0.0031	0.0040	0.0036	0.0044
	DR	-0.0007	-0.0021	0.0007	0.3211							
	TD	0.0000	-0.0001	0.0001	0.9532							
8	NF	0.0008	0.0003	0.0013	0.0012	-0.0030				0.0056	0.0051	0.0062
	DR	-0.0001	-0.0021	0.0018	0.8940							
	TD	0.0001	-0.0001	0.0002	0.3535							
9	NF	0.0007	0.0003	0.0011	0.0005	-0.0046	0.0021	0.0013	0.0033	0.0047	0.0043	0.0052
	DR	0.0015	-0.0001	0.0031	0.0718							
	TD	0.0001	0.0000	0.0002	0.1313							
10	NF	0.0009	0.0004	0.0013	0.0005	-0.0014				0.0054	0.0049	0.0060
	DR	-0.0007	-0.0026	0.0011	0.4494							
	TD	0.0000	-0.0001	0.0002	0.6360							
11	NF	0.0007	0.0003	0.0011	0.0016	-0.0023				0.0048	0.0044	0.0053
	DR	0.0008	-0.0009	0.0025	0.3375							
	TD	0.0000	-0.0001	0.0001	0.8275							
12	NF	0.0010	0.0006	0.0014	0.0000	-0.0047	0.0022	0.0014	0.0035	0.0047	0.0042	0.0052
	DR	0.0008	-0.0008	0.0024	0.3002							

CH	Parameter	Effect size	Lower	Upper	p-value	Estimated intercept	SD	Lower	Upper	SD	Lower	Upper
	TD	0.0001	-0.0001	0.0002	0.4218							
13	NF	0.0009	0.0005	0.0013	0.0000	-0.0038				0.0045	0.0041	0.0050
	DR	0.0012	-0.0004	0.0027	0.1354							
	TD	0.0000	-0.0001	0.0002	0.5621							
14	NF	0.0006	0.0003	0.0010	0.0010	-0.0011				0.0044	0.0040	0.0048
	DR	-0.0004	-0.0019	0.0011	0.6121							
	TD	0.0000	-0.0001	0.0001	0.9852							
15	NF	0.0009	0.0004	0.0013	0.0001	-0.0029				0.0049	0.0044	0.0054
	DR	0.0004	-0.0013	0.0021	0.6140							
	TD	0.0000	-0.0001	0.0001	0.9227							
16	NF	0.0012	0.0008	0.0016	0.0000	-0.0041				0.0044	0.0040	0.0049
	DR	0.0012	-0.0003	0.0027	0.1245							
	TD	0.0000	-0.0002	0.0001	0.5869							
17	NF	0.0010	0.0006	0.0013	0.0000	-0.0032	0.0018	0.0012	0.0029	0.0041	0.0037	0.0046
	DR	0.0009	-0.0005	0.0023	0.2160							
	TD	0.0000	-0.0001	0.0001	0.6127							

NF: natural frequency, DR: damping ratio, TD: task duration, SD: standard deviation, CI: confident interval, df = 9 (the number of period) *22 (the number of participant) -4 (the number of estimated variable) = 194, Significance is identified by CI not including 0 and p-value < 0.05, Gray shade means that identified significance exists only in this 780 nm optical density data, Blue shade means that identified significance exists not only in this 780 nm wavelength data but also in 805 or 830nm wavelength data, Red shade means that identified significance exists in all wavelength data. Blank in random effect column means there is no random effect.

Table S17. Signal amplitude ~ Task duration+ω_n + ζ+(1|subject) , 805 nm optical density data statistical results.

CH	Parameter	Fixed effect				Estimated intercept	Random effect			Error		
		Effect size	CI(95%) Lower	CI(95%) Upper	p-value		SD	CI(95%) Lower	CI(95%) Upper	SD	CI(95%) Lower	CI(95%) Upper
1	NF	0.0009	0.0003	0.0014	0.0036	0.0006				0.0065	0.0059	0.0072
	DR	-0.0009	-0.0032	0.0013	0.4132							
	TD	0.0000	-0.0002	0.0002	0.7919							
2	NF	0.0008	0.0003	0.0012	0.0006	0.0009				0.0051	0.0046	0.0057
	DR	-0.0013	-0.0031	0.0004	0.1329							
	TD	0.0000	-0.0002	0.0001	0.5723							
3	NF	0.0007	0.0001	0.0013	0.0227	0.0013	0.0037	0.0025	0.0056	0.0068	0.0062	0.0076
	DR	-0.0021	-0.0044	0.0003	0.0816							
	TD	0.0000	-0.0002	0.0002	0.8082							
4	NF	0.0008	0.0003	0.0014	0.0037	0.0017				0.0064	0.0058	0.0070
	DR	-0.0014	-0.0036	0.0008	0.2148							
	TD	-0.0001	-0.0002	0.0001	0.4139							
5	NF	0.0011	0.0006	0.0017	0.0001	-0.0012				0.0063	0.0058	0.0070
	DR	-0.0007	-0.0029	0.0015	0.5458							
	TD	0.0000	-0.0002	0.0002	0.8257							
6	NF	0.0007	0.0002	0.0012	0.0083	0.0001	0.0029	0.0019	0.0045	0.0060	0.0054	0.0066
	DR	-0.0012	-0.0033	0.0008	0.2355							
	TD	0.0000	-0.0001	0.0002	0.6457							
7	NF	0.0007	0.0004	0.0011	0.0001	-0.0004	0.0022	0.0015	0.0034	0.0042	0.0038	0.0047
	DR	-0.0010	-0.0025	0.0005	0.1752							
	TD	0.0000	-0.0001	0.0001	0.8244							
8	NF	0.0011	0.0006	0.0016	0.0000	-0.0022				0.0059	0.0054	0.0066
	DR	-0.0008	-0.0029	0.0012	0.4309							
	TD	0.0000	-0.0001	0.0002	0.7573							
9	NF	0.0011	0.0006	0.0015	0.0000	-0.0031	0.0024	0.0015	0.0037	0.0050	0.0045	0.0055
	DR	0.0005	-0.0012	0.0023	0.5286							
	TD	0.0000	-0.0001	0.0002	0.6496							
10	NF	0.0009	0.0004	0.0014	0.0006	-0.0009				0.0057	0.0052	0.0063
	DR	-0.0009	-0.0029	0.0011	0.3719							
	TD	0.0000	-0.0001	0.0002	0.8902							
11	NF	0.0009	0.0004	0.0013	0.0001	-0.0018				0.0051	0.0046	0.0056
	DR	0.0004	-0.0013	0.0022	0.6244							
	TD	0.0000	-0.0002	0.0001	0.8304							
12	NF	0.0013	0.0008	0.0017	0.0000	-0.0041	0.0024	0.0015	0.0038	0.0052	0.0047	0.0058
	DR	0.0004	-0.0014	0.0022	0.6607							
	TD	0.0000	-0.0001	0.0002	0.8992							
13	NF	0.0012	0.0008	0.0017	0.0000	-0.0025				0.0052	0.0047	0.0058
	DR	0.0003	-0.0015	0.0021	0.7305							
	TD	0.0000	-0.0002	0.0001	0.7545							
14	NF	0.0007	0.0003	0.0011	0.0011	-0.0007				0.0048	0.0043	0.0053
	DR	-0.0007	-0.0024	0.0009	0.3883							
	TD	0.0000	-0.0001	0.0001	0.8345							
15	NF	0.0011	0.0006	0.0016	0.0000	-0.0024				0.0052	0.0048	0.0058
	DR	0.0001	-0.0017	0.0019	0.8846							
	TD	0.0000	-0.0002	0.0001	0.5489							
16	NF	0.0014	0.0010	0.0019	0.0000	-0.0037				0.0051	0.0046	0.0057
	DR	0.0010	-0.0007	0.0028	0.2583							

CH	Parameter	Effect size	Lower	Upper	p-value	Estimated intercept	SD	Lower	Upper	SD	Lower	Upper
	TD	-0.0001	-0.0002	0.0001	0.2490							
17	NF	0.0012	0.0008	0.0017	0.0000	-0.0028				0.0052	0.0047	0.0058
	DR	0.0007	-0.0011	0.0025	0.4165							
	TD	-0.0001	-0.0002	0.0001	0.4512							

NF: natural frequency, DR: damping ratio, TD: task duration, SD: standard deviation, CI: confident interval, df = 9 (the number of period) *22 (the number of participant) -4 (the number of estimated variable) = 194, Significance is identified by CI not including 0 and p-value < 0.05, Gray shade means that identified significance exists only in this 805 nm wavelength data, Blue shade means that identified significance exists not only in this 805 nm wavelength data but also in 830nm wavelength data, Red shade means that identified significance exists in all wavelength data. Blank in random effect column means there is no random effect.

Table S18. Signal amplitude ~ Task duration+ω_n + ζ+(1|subject), 830 nm optical density data statistical results.

CH	Parameter	Fixed effect				Estimated intercept	Random effect			Error		
		Effect size	CI(95%) Lower	CI(95%) Upper	p-value		SD	CI(95%) Lower	CI(95%) Upper	SD	CI(95%) Lower	CI(95%) Upper
1	NF	0.0010	0.0003	0.0016	0.0027	0.0008				0.0073	0.0066	0.0080
	DR	-0.0010	-0.0035	0.0015	0.4194							
	TD	0.0000	-0.0002	0.0002	0.6729							
2	NF	0.0010	0.0005	0.0015	0.0001	0.0006				0.0056	0.0050	0.0061
	DR	-0.0013	-0.0032	0.0006	0.1936							
	TD	0.0000	-0.0002	0.0001	0.5622							
3	NF	0.0008	0.0002	0.0014	0.0132	0.0015	0.0036	0.0023	0.0055	0.0071	0.0064	0.0079
	DR	-0.0022	-0.0046	0.0002	0.0766							
	TD	0.0000	-0.0002	0.0002	0.7688							
4	NF	0.0009	0.0003	0.0015	0.0054	0.0021				0.0072	0.0065	0.0080
	DR	-0.0017	-0.0042	0.0008	0.1814							
	TD	-0.0001	-0.0003	0.0001	0.3868							
5	NF	0.0013	0.0007	0.0019	0.0000	-0.0006				0.0068	0.0061	0.0075
	DR	-0.0011	-0.0034	0.0013	0.3653							
	TD	0.0000	-0.0002	0.0001	0.6712							
6	NF	0.0009	0.0003	0.0014	0.0024	0.0002	0.0029	0.0018	0.0046	0.0063	0.0057	0.0070
	DR	-0.0014	-0.0035	0.0008	0.2115							
	TD	0.0000	-0.0002	0.0002	0.8456							
7	NF	0.0008	0.0004	0.0012	0.0001	0.0000	0.0023	0.0015	0.0035	0.0047	0.0042	0.0052
	DR	-0.0014	-0.0030	0.0003	0.0990							
	TD	0.0000	-0.0002	0.0001	0.6281							
8	NF	0.0012	0.0006	0.0018	0.0000	-0.0020				0.0064	0.0058	0.0070
	DR	-0.0010	-0.0032	0.0012	0.3749							
	TD	0.0000	-0.0002	0.0002	0.9366							
9	NF	0.0012	0.0007	0.0017	0.0000	-0.0029	0.0024	0.0015	0.0039	0.0055	0.0050	0.0062
	DR	0.0005	-0.0014	0.0024	0.6090							
	TD	0.0000	-0.0001	0.0002	0.8728							
10	NF	0.0010	0.0005	0.0016	0.0002	-0.0006				0.0063	0.0057	0.0070
	DR	-0.0012	-0.0034	0.0009	0.2603							
	TD	0.0000	-0.0002	0.0002	0.9081							
11	NF	0.0010	0.0005	0.0014	0.0001	-0.0015				0.0055	0.0050	0.0061
	DR	0.0003	-0.0016	0.0022	0.7447							
	TD	0.0000	-0.0002	0.0001	0.7160							
12	NF	0.0014	0.0009	0.0019	0.0000	-0.0040	0.0025	0.0016	0.0040	0.0056	0.0050	0.0062
	DR	0.0004	-0.0015	0.0023	0.6864							
	TD	0.0000	-0.0002	0.0001	0.8787							
13	NF	0.0013	0.0008	0.0018	0.0000	-0.0022				0.0058	0.0052	0.0064
	DR	0.0002	-0.0018	0.0022	0.8604							
	TD	0.0000	-0.0002	0.0001	0.6005							
14	NF	0.0009	0.0004	0.0013	0.0003	-0.0005				0.0052	0.0047	0.0058
	DR	-0.0010	-0.0028	0.0008	0.2671							
	TD	0.0000	-0.0002	0.0001	0.6107							
15	NF	0.0012	0.0007	0.0017	0.0000	-0.0025				0.0056	0.0051	0.0062
	DR	0.0001	-0.0018	0.0020	0.9073							
	TD	0.0000	-0.0002	0.0001	0.5335							
16	NF	0.0015	0.0010	0.0020	0.0000	-0.0035				0.0055	0.0049	0.0060
	DR	0.0009	-0.0010	0.0027	0.3692							
	TD	-0.0001	-0.0002	0.0001	0.2030							
17	NF	0.0013	0.0008	0.0018	0.0000	-0.0025				0.0057	0.0052	0.0063
	DR	0.0003	-0.0016	0.0023	0.7472							
	TD	-0.0001	-0.0002	0.0001	0.4381							

NF: natural frequency, DR: damping ratio, TD: task duration, SD: standard deviation, CI: confident interval, df = 9 (the number of period) *22 (the number of participant) -4 (the number of estimated variable) = 194, Significance is identified by CI not including 0 and p-value < 0.05, Gray shade means that identified significance exists only in this 830 nm wavelength data. Blue shade means that identified significance exists not only in this 830 nm wavelength data but also in 805nm wavelength data, Red shade means that identified significance exists in all wavelength data.

Table S19. Beta ~ State+(1|subject) , 780 nm optical density data statistical results.

CH	Parameter	Fixed effect				Estimated intercept	Random effect			Error		
		Effect size	CI(95%)		p-value		SD	CI(95%)		SD	CI(95%)	
			Lower	Upper				Lower	Upper		Lower	Upper
1	State	0.0029	0.0014	0.0044	0.0001	-0.0015				0.0074	0.0069	0.0080
2	State	0.0016	0.0004	0.0027	0.0070	-0.0008				0.0057	0.0053	0.0061
3	State	0.0004	-0.0017	0.0025	0.7363	-0.0002				0.0106	0.0099	0.0114
4	State	0.0029	0.0015	0.0043	0.0000	-0.0014				0.0070	0.0065	0.0075
5	State	0.0012	-0.0002	0.0025	0.0913	-0.0006				0.0069	0.0064	0.0074
6	State	0.0000	-0.0018	0.0018	0.9896	0.0000				0.0092	0.0086	0.0099
7	State	0.0010	-0.0001	0.0021	0.0694	-0.0005				0.0056	0.0052	0.0060
8	State	0.0000	-0.0013	0.0013	0.9636	0.0000				0.0067	0.0062	0.0071
9	State	0.0002	-0.0011	0.0014	0.8093	-0.0001				0.0065	0.0061	0.0070
10	State	0.0018	0.0003	0.0033	0.0218	-0.0009				0.0077	0.0072	0.0083
11	State	0.0017	0.0005	0.0028	0.0039	-0.0008				0.0058	0.0054	0.0062
12	State	-0.0006	-0.0019	0.0007	0.3515	0.0003				0.0064	0.0059	0.0068
13	State	0.0012	0.0001	0.0023	0.0405	-0.0006				0.0058	0.0054	0.0062
14	State	0.0010	-0.0001	0.0021	0.0813	-0.0005				0.0055	0.0051	0.0059
15	State	0.0005	-0.0007	0.0017	0.4373	-0.0002				0.0060	0.0056	0.0065
16	State	0.0005	-0.0007	0.0016	0.4028	-0.0002				0.0058	0.0054	0.0062
17	State	0.0009	-0.0003	0.0020	0.1276	-0.0004				0.0057	0.0053	0.0061

SD: standard deviation, CI: confident interval, the state consists of the rest state and task state, The value of the rest state is 0, and the value of the task state is 1, df = 18 (the number of period) *22 (the number of participant) -2 (the number of estimated variable) = 394, Significance is identified by CI not including 0 and p-value < 0.05, Red shade means that identified significance exists in all wavelength data. There is no random effect.

Table S20. Beta ~ State+(1|subject) , 805 nm optical density data statistical results.

CH	Parameter	Fixed effect				Estimated intercept	Random effect			Error		
		Effect size	CI(95%)		p-value		SD	CI(95%)		SD	CI(95%)	
			Lower	Upper				Lower	Upper		Lower	Upper
1	State	0.0044	0.0028	0.0059	0.0000	-0.0022				0.0079	0.0074	0.0085
2	State	0.0035	0.0023	0.0047	0.0000	-0.0017				0.0061	0.0057	0.0065
3	State	0.0025	0.0004	0.0046	0.0207	-0.0013				0.0107	0.0100	0.0114
4	State	0.0046	0.0030	0.0061	0.0000	-0.0023				0.0078	0.0073	0.0084
5	State	0.0032	0.0017	0.0047	0.0000	-0.0015				0.0076	0.0071	0.0081
6	State	0.0031	0.0014	0.0049	0.0005	-0.0016				0.0088	0.0083	0.0095
7	State	0.0015	0.0003	0.0027	0.0138	-0.0007				0.0060	0.0056	0.0064
8	State	0.0012	-0.0002	0.0026	0.0908	-0.0006				0.0072	0.0067	0.0077
9	State	0.0021	0.0008	0.0035	0.0023	-0.0010				0.0070	0.0065	0.0075
10	State	0.0022	0.0007	0.0038	0.0055	-0.0011				0.0080	0.0075	0.0086
11	State	0.0027	0.0015	0.0040	0.0000	-0.0013				0.0062	0.0058	0.0067
12	State	0.0005	-0.0009	0.0019	0.4627	-0.0002				0.0071	0.0066	0.0076
13	State	0.0029	0.0016	0.0042	0.0000	-0.0014				0.0065	0.0061	0.0070
14	State	0.0012	0.0001	0.0024	0.0401	-0.0006				0.0059	0.0055	0.0063
15	State	0.0013	0.0000	0.0027	0.0447	-0.0007				0.0067	0.0062	0.0071
16	State	0.0017	0.0004	0.0030	0.0109	-0.0008				0.0066	0.0061	0.0070
17	State	0.0023	0.0011	0.0036	0.0004	-0.0011				0.0065	0.0060	0.0069

SD: standard deviation, CI: confident interval, the state consists of the rest state and task state, The value of the rest state is 0, and the value of the task state is 1, df = 18 (the number of period) *22 (the number of participant) -2 (the number of estimated variable) = 394, Significance is identified by CI not including 0 and p-value < 0.05, Blue shade means that identified significance exists not only in this 805 nm wavelength data but also in 830nm wavelength data, Red shade means that identified significance exists in all wavelength data. There is no random effect.

Table S21. Beta ~ State+(1|subject) , 830 nm optical density data statistical results.

CH	Parameter	Fixed effect				Estimated intercept	Random effect			Error		
		Effect size	CI(95%)		p-value		SD	CI(95%)		SD	CI(95%)	
			Lower	Upper				Lower	Upper		Lower	Upper
1	State	0.0051	0.0034	0.0069	0.0000	-0.0025				0.0088	0.0082	0.0094
2	State	0.0041	0.0028	0.0054	0.0000	-0.0020				0.0066	0.0062	0.0071
3	State	0.0032	0.0010	0.0053	0.0038	-0.0016				0.0108	0.0101	0.0116
4	State	0.0052	0.0034	0.0069	0.0000	-0.0025				0.0088	0.0082	0.0094
5	State	0.0040	0.0024	0.0056	0.0000	-0.0020				0.0080	0.0075	0.0086
6	State	0.0037	0.0019	0.0055	0.0001	-0.0018				0.0091	0.0085	0.0098
7	State	0.0019	0.0006	0.0032	0.0043	-0.0009				0.0066	0.0061	0.0070

8	State	0.0016	0.0001	0.0031	0.0364	-0.0008				0.0076	0.0071	0.0082
9	State	0.0027	0.0012	0.0042	0.0004	-0.0013				0.0076	0.0071	0.0081
10	State	0.0028	0.0011	0.0044	0.0013	-0.0014				0.0085	0.0079	0.0091
11	State	0.0031	0.0018	0.0045	0.0000	-0.0015				0.0067	0.0063	0.0072
12	State	0.0008	-0.0007	0.0024	0.2671	-0.0004				0.0076	0.0071	0.0081
13	State	0.0032	0.0018	0.0046	0.0000	-0.0016				0.0071	0.0066	0.0076
14	State	0.0015	0.0002	0.0028	0.0227	-0.0007				0.0064	0.0060	0.0069
15	State	0.0016	0.0002	0.0030	0.0245	-0.0008				0.0071	0.0066	0.0076
16	State	0.0021	0.0007	0.0034	0.0033	-0.0010				0.0070	0.0065	0.0075
17	State	0.0025	0.0012	0.0039	0.0003	-0.0012				0.0070	0.0065	0.0075

SD: standard deviation, CI: confident interval, the state consists of the rest state and task state, The value of the rest state is 0, and the value of the task state is 1, df = 18 (the number of period) *22 (the number of participant) -2 (the number of estimated variable) = 394, Significance is identified by CI not including 0 and p-value < 0.05, Gray shade means that identified significance exists only in this 830 nm wavelength data, Blue shade means that identified significance exists not only in this 830 nm wavelength data but also in 805nm wavelength data, Red shade means that identified significance exists in all wavelength density data. There is no random effect.

Table S22. Beta~ Rest duration+ (1|subject) , 780 nm optical density data statistical results.

CH	Parameter	Fixed effect				Estimated intercept	Random effect			Error		
		Effect size*	CI(95%)		p-value		SD	CI(95%)		SD	CI(95%)	
			Lower*	Upper*				Lower	Upper		Lower	Upper
1	RD	-0.1490	-0.3590	0.0611	0.1635	-0.0001				0.0077	0.0070	0.0085
2	RD	-0.0895	-0.2517	0.0727	0.2781	0.0000				0.0060	0.0054	0.0066
3	RD	-0.1029	-0.4290	0.2232	0.5344	0.0007				0.0120	0.0109	0.0133
4	RD	-0.0823	-0.2824	0.1178	0.4184	-0.0007				0.0074	0.0067	0.0081
5	RD	-0.1165	-0.3056	0.0726	0.2257	0.0005				0.0070	0.0063	0.0077
6	RD	-0.1702	-0.4536	0.1133	0.2378	0.0015				0.0104	0.0095	0.0115
7	RD	-0.0655	-0.2265	0.0955	0.4233	0.0001				0.0059	0.0054	0.0065
8	RD	-0.1453	-0.3248	0.0342	0.1119	0.0013				0.0066	0.0060	0.0073
9	RD	-0.1662	-0.3503	0.0178	0.0765	0.0014				0.0068	0.0061	0.0075
10	RD	-0.1420	-0.3795	0.0954	0.2395	0.0004				0.0087	0.0079	0.0097
11	RD	-0.0971	-0.2559	0.0617	0.2294	0.0000				0.0059	0.0053	0.0065
12	RD	-0.1205	-0.2947	0.0538	0.1743	0.0014				0.0064	0.0058	0.0071
13	RD	-0.1271	-0.2913	0.0371	0.1285	0.0006				0.0061	0.0055	0.0067
14	RD	-0.0621	-0.2204	0.0961	0.4396	0.0001				0.0058	0.0053	0.0064
15	RD	-0.0795	-0.2474	0.0885	0.3518	0.0005				0.0062	0.0056	0.0068
16	RD	-0.0446	-0.2065	0.1172	0.5872	0.0002				0.0060	0.0054	0.0066
17	RD	-0.0455	-0.2059	0.1148	0.5759	0.0000				0.0059	0.0054	0.0065

RD: rest duration, $\bullet^{*}: \times 10^{-3}$, SD: standard deviation, CI: confident interval, Beta value calculated from the rest state only, df = 9 (the number of period) *22 (the number of participant) -2 (the number of estimated variable) = 196, Significance is identified by CI not including 0 and p-value < 0.05, There is no random effect.

Table S23. Beta~ Rest duration+ (1|subject) , 805 nm optical density data statistical results.

CH	Parameter	Fixed effect				Estimated intercept	Random effect			Error		
		Effect size*	CI(95%)		p-value		SD	CI(95%)		SD	CI(95%)	
			Lower*	Upper*				Lower	Upper		Lower	Upper
1	RD	-0.1105	-0.3339	0.1129	0.3304	-0.0012				0.0082	0.0075	0.0091
2	RD	-0.0761	-0.2408	0.0886	0.3633	-0.0010				0.0061	0.0055	0.0067
3	RD	-0.1089	-0.4365	0.2188	0.5131	-0.0003				0.0121	0.0109	0.0133
4	RD	-0.0563	-0.2771	0.1645	0.6155	-0.0018				0.0081	0.0074	0.0090
5	RD	-0.1033	-0.3085	0.1019	0.3221	-0.0006				0.0076	0.0069	0.0083
6	RD	-0.1702	-0.4353	0.0949	0.2070	0.0000				0.0098	0.0089	0.0108
7	RD	-0.0678	-0.2390	0.1034	0.4356	-0.0001				0.0063	0.0057	0.0070
8	RD	-0.1285	-0.3231	0.0662	0.1946	0.0006				0.0072	0.0065	0.0079
9	RD	-0.1379	-0.3340	0.0582	0.1672	0.0002				0.0072	0.0065	0.0080
10	RD	-0.1202	-0.3661	0.1257	0.3362	0.0000				0.0091	0.0082	0.0100
11	RD	-0.0913	-0.2637	0.0811	0.2977	-0.0005				0.0064	0.0058	0.0070
12	RD	-0.1079	-0.3042	0.0883	0.2794	0.0007				0.0072	0.0066	0.0080
13	RD	-0.1068	-0.2873	0.0737	0.2449	-0.0005				0.0067	0.0060	0.0073
14	RD	-0.0626	-0.2308	0.1055	0.4635	0.0000				0.0062	0.0056	0.0068
15	RD	-0.0506	-0.2396	0.1383	0.5978	-0.0002				0.0070	0.0063	0.0077
16	RD	-0.0248	-0.2068	0.1572	0.7886	-0.0006				0.0067	0.0061	0.0074
17	RD	-0.0592	-0.2371	0.1187	0.5123	-0.0006				0.0066	0.0059	0.0072

RD: rest duration, $\bullet^{*}: \times 10^{-3}$, SD: standard deviation, CI: confident interval, Beta value calculated from the rest state only, df = 9 (the number of period) *22 (the number of participant) -2 (the number of estimated variable) = 196, Significance is identified by CI not including 0 and p-value < 0.05, There is no random effect.

Table S24. Beta~ Rest duration+ (1|subject) , 830 nm optical density data statistical results.

CH	Parameter	Fixed effect				Estimated intercept	Random effect			Error		
		Effect size*	CI(95%)		p-value		SD	CI(95%)		SD	CI(95%)	
			Lower*	Upper*				Lower	Upper		Lower	Upper
1	RD	-0.1143	-0.3581	0.1295	0.3564	-0.0015				0.0090	0.0081	0.0099
2	RD	-0.0884	-0.2670	0.0902	0.3304	-0.0012				0.0066	0.0060	0.0073
3	RD	-0.1198	-0.4495	0.2099	0.4746	-0.0005				0.0121	0.0110	0.0134
4	RD	-0.0565	-0.3031	0.1902	0.6521	-0.0020				0.0091	0.0082	0.0100
5	RD	-0.1042	-0.3200	0.1116	0.3421	-0.0010				0.0080	0.0072	0.0088
6	RD	-0.1679	-0.4398	0.1041	0.2249	-0.0003				0.0100	0.0091	0.0111
7	RD	-0.0635	-0.2500	0.1230	0.5026	-0.0004				0.0069	0.0062	0.0076
8	RD	-0.1191	-0.3237	0.0854	0.2521	0.0003				0.0075	0.0068	0.0083
9	RD	-0.1356	-0.3492	0.0779	0.2118	-0.0001				0.0079	0.0071	0.0087
10	RD	-0.1192	-0.3720	0.1336	0.3535	-0.0003				0.0093	0.0084	0.0103
11	RD	-0.0891	-0.2764	0.0982	0.3492	-0.0007				0.0069	0.0063	0.0076
12	RD	-0.0941	-0.3034	0.1152	0.3765	0.0004				0.0077	0.0070	0.0085
13	RD	-0.0944	-0.2877	0.0990	0.3370	-0.0007				0.0071	0.0065	0.0079
14	RD	-0.0477	-0.2290	0.1336	0.6044	-0.0003				0.0067	0.0061	0.0074
15	RD	-0.0510	-0.2535	0.1515	0.6199	-0.0003				0.0075	0.0068	0.0082
16	RD	-0.0210	-0.2126	0.1707	0.8295	-0.0008				0.0071	0.0064	0.0078
17	RD	-0.0606	-0.2504	0.1292	0.5296	-0.0007				0.0070	0.0063	0.0077

RD: rest duration, $\bullet^{*}: \times 10^{-3}$, SD: standard deviation, CI: confident interval, Beta value calculated from the rest state only, df = 9 (the number of period) *22 (the number of participant) -2 (the number of estimated variable) = 196, Significance is identified by CI not including 0 and p-value < 0.05, There is no random effect.

Table S25. Beta ~ Task duration+ω_n + ζ+(1|subject) , 780 nm optical density data statistical results.

CH	Parameter	Fixed effect				Estimated intercept	Random effect			Error		
		Effect size	CI(95%)		p-value		SD	CI(95%)		SD	CI(95%)	
			Lower	Upper				Lower	Upper		Lower	Upper
1	NF	0.0006	0.0000	0.0013	0.0383	-0.0010				0.0070	0.0063	0.0077
	DR	0.0000	-0.0024	0.0024	0.9951							
	TD	0.0000	-0.0001	0.0002	0.6181							
2	NF	0.0005	0.0000	0.0009	0.0480	-0.0004				0.0053	0.0048	0.0059
	DR	-0.0005	-0.0023	0.0014	0.6186							
	TD	0.0000	-0.0001	0.0002	0.7165							
3	NF	0.0005	-0.0002	0.0012	0.1427	-0.0004	0.0047	0.0032	0.0068	0.0076	0.0068	0.0084
	DR	-0.0012	-0.0038	0.0015	0.3835							
	TD	0.0000	-0.0002	0.0002	0.8075							
4	NF	0.0008	0.0002	0.0014	0.0055	-0.0001				0.0064	0.0058	0.0071
	DR	-0.0006	-0.0028	0.0016	0.6039							
	TD	0.0000	-0.0002	0.0002	0.7812							
5	NF	0.0009	0.0003	0.0015	0.0024	-0.0027				0.0066	0.0060	0.0073
	DR	0.0002	-0.0021	0.0024	0.8900							
	TD	0.0000	-0.0001	0.0002	0.6263							
6	NF	0.0004	-0.0001	0.0010	0.1296	-0.0023	0.0042	0.0029	0.0061	0.0064	0.0058	0.0071
	DR	-0.0003	-0.0025	0.0019	0.7847							
	TD	0.0001	-0.0001	0.0003	0.1639							
7	NF	0.0008	0.0004	0.0012	0.0002	-0.0009	0.0023	0.0015	0.0035	0.0045	0.0041	0.0050
	DR	-0.0008	-0.0023	0.0008	0.3209							
	TD	0.0000	-0.0001	0.0001	0.9543							
8	NF	0.0009	0.0004	0.0015	0.0012	-0.0035				0.0065	0.0058	0.0071
	DR	-0.0002	-0.0024	0.0021	0.8934							
	TD	0.0001	-0.0001	0.0003	0.3534							
9	NF	0.0008	0.0004	0.0013	0.0005	-0.0053	0.0024	0.0015	0.0038	0.0054	0.0049	0.0060
	DR	0.0017	-0.0002	0.0036	0.0719							
	TD	0.0001	0.0000	0.0003	0.1311							
10	NF	0.0010	0.0004	0.0015	0.0005	-0.0016				0.0062	0.0056	0.0069
	DR	-0.0008	-0.0030	0.0013	0.4498							
	TD	0.0000	-0.0001	0.0002	0.6344							
11	NF	0.0008	0.0003	0.0013	0.0016	-0.0026				0.0055	0.0050	0.0061
	DR	0.0009	-0.0010	0.0028	0.3376							
	TD	0.0000	-0.0001	0.0002	0.8279							
12	NF	0.0012	0.0007	0.0016	0.0000	-0.0054	0.0025	0.0016	0.0040	0.0053	0.0048	0.0059
	DR	0.0010	-0.0009	0.0028	0.3004							
	TD	0.0001	-0.0001	0.0002	0.4217							
13	NF	0.0010	0.0006	0.0015	0.0000	-0.0043				0.0052	0.0047	0.0057
	DR	0.0014	-0.0004	0.0031	0.1354							
	TD	0.0000	-0.0001	0.0002	0.5615							

14	NF	0.0007	0.0003	0.0012	0.0010	-0.0013				0.0050	0.0045	0.0055
	DR	-0.0004	-0.0022	0.0013	0.6119							
	TD	0.0000	-0.0001	0.0001	0.9852							
15	NF	0.0010	0.0005	0.0015	0.0001	-0.0033				0.0056	0.0051	0.0062
	DR	0.0005	-0.0014	0.0024	0.6139							
	TD	0.0000	-0.0001	0.0002	0.9232							
16	NF	0.0013	0.0009	0.0018	0.0000	-0.0047				0.0051	0.0046	0.0056
	DR	0.0014	-0.0004	0.0031	0.1247							
	TD	0.0000	-0.0002	0.0001	0.5871							
17	NF	0.0011	0.0007	0.0015	0.0000	-0.0036	0.0021	0.0013	0.0033	0.0047	0.0042	0.0052
	DR	0.0010	-0.0006	0.0026	0.2164							
	TD	0.0000	-0.0002	0.0001	0.6128							

NF: natural frequency, DR: damping ratio, TD: task duration, SD: standard deviation, CI: confident interval, df = 9 (the number of period) *22 (the number of participant) -4 (the number of estimated variable) = 194, Significance is identified by CI not including 0 and p-value < 0.05, Gray shade means that identified significance exists only in this 780 nm optical density data, Blue shade means that identified significance exists not only in this 780 nm wavelength data but also in 805 or 830nm wavelength data, Red shade means that identified significance exists in all wavelength data. Blank in random effect column means there is no random effect.

Table S26. Beta ~ Task duration+ω_n + ζ+(1|subject), 805 nm optical density data statistical results.

CH	Parameter	Fixed effect				Estimated intercept	Random effect			Error		
		Effect size	CI(95%)		p-value		SD	CI(95%)		SD	CI(95%)	
			Lower	Upper				Lower	Upper		Lower	Upper
1	NF	0.0010	0.0003	0.0016	0.0036	0.0006				0.0075	0.0068	0.0082
	DR	-0.0011	-0.0036	0.0015	0.4125							
	TD	0.0000	-0.0002	0.0002	0.7918							
2	NF	0.0009	0.0004	0.0014	0.0005	0.0010				0.0059	0.0053	0.0065
	DR	-0.0015	-0.0036	0.0005	0.1328							
	TD	0.0000	-0.0002	0.0001	0.5714							
3	NF	0.0008	0.0001	0.0015	0.0225	0.0015	0.0042	0.0028	0.0064	0.0078	0.0070	0.0087
	DR	-0.0024	-0.0051	0.0003	0.0815							
	TD	0.0000	-0.0002	0.0002	0.8098							
4	NF	0.0009	0.0003	0.0016	0.0036	0.0019				0.0073	0.0066	0.0080
	DR	-0.0016	-0.0041	0.0009	0.2145							
	TD	-0.0001	-0.0003	0.0001	0.4129							
5	NF	0.0013	0.0007	0.0019	0.0001	-0.0013				0.0073	0.0066	0.0080
	DR	-0.0008	-0.0033	0.0017	0.5451							
	TD	0.0000	-0.0002	0.0002	0.8250							
6	NF	0.0008	0.0002	0.0014	0.0082	0.0001	0.0033	0.0022	0.0052	0.0069	0.0062	0.0076
	DR	-0.0014	-0.0038	0.0009	0.2353							
	TD	0.0000	-0.0001	0.0002	0.6442							
7	NF	0.0008	0.0004	0.0013	0.0001	-0.0004	0.0025	0.0017	0.0038	0.0048	0.0044	0.0054
	DR	-0.0011	-0.0028	0.0005	0.1753							
	TD	0.0000	-0.0001	0.0001	0.8253							
8	NF	0.0013	0.0007	0.0019	0.0000	-0.0025				0.0068	0.0062	0.0075
	DR	-0.0009	-0.0033	0.0014	0.4307							
	TD	0.0000	-0.0002	0.0002	0.7577							
9	NF	0.0012	0.0007	0.0017	0.0000	-0.0035	0.0027	0.0017	0.0042	0.0057	0.0051	0.0063
	DR	0.0006	-0.0013	0.0026	0.5287							
	TD	0.0000	-0.0001	0.0002	0.6491							
10	NF	0.0010	0.0004	0.0016	0.0006	-0.0010				0.0066	0.0059	0.0072
	DR	-0.0010	-0.0033	0.0012	0.3720							
	TD	0.0000	-0.0002	0.0002	0.8883							
11	NF	0.0010	0.0005	0.0015	0.0001	-0.0020				0.0059	0.0053	0.0065
	DR	0.0005	-0.0015	0.0025	0.6241							
	TD	0.0000	-0.0002	0.0001	0.8297							
12	NF	0.0015	0.0009	0.0020	0.0000	-0.0046	0.0027	0.0017	0.0043	0.0059	0.0054	0.0066
	DR	0.0005	-0.0016	0.0025	0.6608							
	TD	0.0000	-0.0002	0.0002	0.8995							
13	NF	0.0014	0.0009	0.0019	0.0000	-0.0028				0.0060	0.0054	0.0066
	DR	0.0004	-0.0017	0.0024	0.7303							
	TD	0.0000	-0.0002	0.0001	0.7550							
14	NF	0.0008	0.0003	0.0013	0.0011	-0.0008				0.0055	0.0049	0.0060
	DR	-0.0008	-0.0027	0.0011	0.3882							
	TD	0.0000	-0.0002	0.0001	0.8353							
15	NF	0.0013	0.0007	0.0018	0.0000	-0.0028				0.0060	0.0054	0.0066
	DR	0.0002	-0.0019	0.0022	0.8840							
	TD	-0.0001	-0.0002	0.0001	0.5485							
16	NF	0.0016	0.0011	0.0021	0.0000	-0.0042				0.0059	0.0053	0.0065
	DR	0.0012	-0.0009	0.0032	0.2581							
	TD	-0.0001	-0.0003	0.0001	0.2491							
17	NF	0.0014	0.0009	0.0019	0.0000	-0.0033				0.0060	0.0054	0.0066
	DR	0.0008	-0.0012	0.0029	0.4170							
	TD	-0.0001	-0.0002	0.0001	0.4527							

NF: natural frequency, DR: damping ratio, TD: task duration, SD: standard deviation, CI: confident interval, df = 9 (the number of period) *22 (the

number of participant) -4 (the number of estimated variable) = 194, Significance is identified by CI not including 0 and p-value < 0.05, Gray shade means that identified significance exists only in this 805 nm wavelength data, Blue shade means that identified significance exists not only in this 805 nm wavelength data but also in 830nm wavelength data, Red shade means that identified significance exists in all wavelength data. Blank in random effect column means there is no random effect.

Table S27. Beta ~ Task duration+ω_n + ζ+(1|subject), 830 nm optical density data statistical results.

CH	Parameter	Fixed effect				Estimated intercept	Random effect			Error		
		Effect size	CI(95%)		p-value		SD	CI(95%)		SD	CI(95%)	
			Lower	Upper				Lower	Upper		Lower	Upper
1	NF	0.0011	0.0004	0.0018	0.0027	0.0009				0.0083	0.0075	0.0092
	DR	-0.0012	-0.0040	0.0017	0.4185							
	TD	0.0000	-0.0003	0.0002	0.6725							
2	NF	0.0011	0.0006	0.0017	0.0001	0.0007				0.0064	0.0058	0.0070
	DR	-0.0014	-0.0036	0.0007	0.1934							
	TD	-0.0001	-0.0002	0.0001	0.5622							
3	NF	0.0009	0.0002	0.0016	0.0131	0.0017	0.0041	0.0027	0.0063	0.0081	0.0073	0.0090
	DR	-0.0025	-0.0053	0.0003	0.0765							
	TD	0.0000	-0.0003	0.0002	0.7703							
4	NF	0.0010	0.0003	0.0017	0.0053	0.0025				0.0082	0.0075	0.0091
	DR	-0.0019	-0.0048	0.0009	0.1812							
	TD	-0.0001	-0.0003	0.0001	0.3859							
5	NF	0.0015	0.0008	0.0022	0.0000	-0.0007				0.0077	0.0070	0.0085
	DR	-0.0012	-0.0039	0.0014	0.3654							
	TD	0.0000	-0.0003	0.0002	0.6711							
6	NF	0.0010	0.0004	0.0016	0.0024	0.0003	0.0033	0.0021	0.0052	0.0072	0.0065	0.0080
	DR	-0.0016	-0.0040	0.0009	0.2112							
	TD	0.0000	-0.0002	0.0002	0.8439							
7	NF	0.0009	0.0005	0.0014	0.0001	0.0000	0.0026	0.0017	0.0040	0.0054	0.0049	0.0060
	DR	-0.0016	-0.0034	0.0003	0.0989							
	TD	0.0000	-0.0002	0.0001	0.6286							
8	NF	0.0014	0.0007	0.0020	0.0000	-0.0022				0.0073	0.0066	0.0081
	DR	-0.0011	-0.0036	0.0014	0.3748							
	TD	0.0000	-0.0002	0.0002	0.9367							
9	NF	0.0013	0.0008	0.0019	0.0000	-0.0033	0.0028	0.0018	0.0045	0.0063	0.0057	0.0070
	DR	0.0006	-0.0016	0.0027	0.6092							
	TD	0.0000	-0.0002	0.0002	0.8724							
10	NF	0.0012	0.0006	0.0018	0.0002	-0.0007				0.0072	0.0065	0.0080
	DR	-0.0014	-0.0039	0.0011	0.2608							
	TD	0.0000	-0.0002	0.0002	0.9089							
11	NF	0.0011	0.0005	0.0016	0.0001	-0.0017				0.0063	0.0057	0.0070
	DR	0.0004	-0.0018	0.0025	0.7447							
	TD	0.0000	-0.0002	0.0001	0.7153							
12	NF	0.0016	0.0010	0.0021	0.0000	-0.0046	0.0029	0.0018	0.0046	0.0064	0.0058	0.0071
	DR	0.0004	-0.0017	0.0026	0.6864							
	TD	0.0000	-0.0002	0.0002	0.8788							
13	NF	0.0015	0.0009	0.0020	0.0000	-0.0025				0.0066	0.0060	0.0073
	DR	0.0002	-0.0021	0.0025	0.8605							
	TD	0.0000	-0.0002	0.0001	0.6013							
14	NF	0.0010	0.0005	0.0015	0.0003	-0.0006				0.0060	0.0054	0.0066
	DR	-0.0012	-0.0032	0.0009	0.2669							
	TD	0.0000	-0.0002	0.0001	0.6112							
15	NF	0.0014	0.0008	0.0019	0.0000	-0.0028				0.0064	0.0058	0.0071
	DR	0.0001	-0.0021	0.0023	0.9067							
	TD	-0.0001	-0.0002	0.0001	0.5328							
16	NF	0.0017	0.0012	0.0023	0.0000	-0.0040				0.0062	0.0057	0.0069
	DR	0.0010	-0.0012	0.0031	0.3692							
	TD	-0.0001	-0.0003	0.0001	0.2031							
17	NF	0.0015	0.0009	0.0021	0.0000	-0.0029				0.0066	0.0059	0.0072
	DR	0.0004	-0.0019	0.0026	0.7469							
	TD	-0.0001	-0.0003	0.0001	0.4383							

NF: natural frequency, DR: damping ratio, TD: task duration, SD: standard deviation, CI: confident interval, df = 9 (the number of period) *22 (the number of participant) -4 (the number of estimated variable) = 194, Significance is identified by CI not including 0 and p-value < 0.05, Gray shade means that identified significance exists only in this 830 nm wavelength data. Blue shade means that identified significance exists not only in this 830 nm wavelength data but also in 805nm wavelength data, Red shade means that identified significance exists in all wavelength data.

Table S28. Mechanical impedance conditions.

		Damping ratio		
	ζ / ω_n	0.5	1	1.5
Natural frequency	1	#1	#2	#3
	3	#4	#5	#6
	5	#6	#7	#9

Table S29. Mechanical impedance condition orders of participants.

Participant	Mechanical impedance condition order								
kmj(M)	3	8	7	2	1	6	5	9	4
wjh(F)	3	1	5	2	7	9	8	4	6
ksj_1(M)	1	9	8	3	5	4	7	6	2
kjs(M)	2	9	5	8	4	3	6	1	7
lcj(M)	1	3	7	4	6	8	2	9	5
cgy(F)	6	8	4	7	5	2	3	9	1
cpj(M)	6	3	1	5	9	4	8	7	2
lkj(F)	2	8	1	6	3	9	5	7	4
lje(F)	5	2	4	7	3	9	8	6	1
jnc(M)	3	1	6	7	2	4	9	5	8
sjh(M)	4	1	3	5	2	8	9	7	6
jsj(F)	6	7	3	1	2	5	9	4	8
hjw(M)	2	1	4	3	9	8	7	5	6
kjh(M)	6	8	1	5	9	3	2	4	7
cjh(M)	4	7	6	2	9	8	5	1	3
nse(M)	5	2	9	4	8	1	6	7	3
sey(F)	7	2	9	5	3	1	6	8	4
kjc(M)	1	6	5	3	4	8	9	2	7
hmc(M)	6	5	3	1	7	8	9	4	2
ksj_2(M)	2	9	3	5	1	8	7	6	4
ces(F)	3	9	4	7	2	5	1	8	6
sah(F)	7	6	2	3	1	9	4	5	8

M: male, F: female, ksj_1 and ksj_2 are different participants. Mechanical impedance conditions were conducted at the task period.

Table S30. Summary of results of the previous study.

Motion \ Neuron group	**Group A**	**Group B**
Elbow extension torque	Discharge rate decreases. (Case 1.)	Discharge rate increases. (Case 2.)
Elbow flexion torque	Discharge rate increases. (Case 3.)	Discharge rate decreases. (Case 4.)
